# Genome-wide clonal variability in European pear “Rocha” using high-throughput sequencing

**DOI:** 10.1093/hr/uhac111

**Published:** 2022-05-17

**Authors:** Octávio Serra, Rui Maia de Sousa, Joana Bagoin Guimarães, José Matos, Patricia Vicente, Miguel Leão de Sousa, Fernanda Simões

**Affiliations:** Instituto Nacional de Investigação Agrária e Veterinária, I.P., Banco Português de Germoplasma Vegetal (BPGV), Quinta de S. José, S. Pedro de Merelim 4700-859 Braga, Portugal; Instituto Nacional de Investigação Agrária e Veterinária, I.P., Estação Nacional de Fruticultura Vieira Natividade (ENFVN), Estrada de Leiria 2460-059 Alcobaça, Portugal; Instituto Nacional de Investigação Agrária e Veterinária, I.P., Quinta do Marquês, 2780-159 Oeiras, Portugal; Instituto Nacional de Investigação Agrária e Veterinária, I.P., Quinta do Marquês, 2780-159 Oeiras, Portugal; Centre for Ecology, Evolution and Environmental Changes, Faculdade de Ciências, Universidade de Lisboa, Campo Grande, 1749-016 Lisboa, Portugal; Instituto Nacional de Investigação Agrária e Veterinária, I.P., Estação Nacional de Fruticultura Vieira Natividade (ENFVN), Estrada de Leiria 2460-059 Alcobaça, Portugal; Instituto Nacional de Investigação Agrária e Veterinária, I.P., Estação Nacional de Fruticultura Vieira Natividade (ENFVN), Estrada de Leiria 2460-059 Alcobaça, Portugal; Instituto Nacional de Investigação Agrária e Veterinária, I.P., Quinta do Marquês, 2780-159 Oeiras, Portugal

## Abstract

Pears (*Pyrus*) are one of the most economically important fruits worldwide. The *Pyrus* genus is characterized by a high degree of genetic variability between species and interspecific hybrids, and several studies have been performed to assess this variability for both cultivated and wild accessions. These studies have mostly been limited by the resolving power of traditional molecular markers, although in the recent past the availability of reference genome sequences or SNP arrays for pear have enhanced the capability of high-resolution genomics studies. These tools can also be applied to better understand the intra-varietal (or clonal) variability in pear. Here we report the first high resolution genomics analysis of a pear clonal population using whole genome sequencing (WGS). Results showed unique signatures for the accumulation of mutations and transposable element insertions in each clone, which are likely related to their history of propagation and cultivation. The nucleotide diversity remained low in the clonal collection with the exception of few genomic windows, suggesting that balancing selection may be occurring. These windows included mainly genes related to plant fertility. Regions with higher mutational load were partially associated with transcription factors, probably reflecting the distinctive phenotypes in the collection. The annotation of variants also revealed the theoretical disruption of relevant genes in pear. Taken together, the results from this study show that pear clones accumulate mutations differently, and that those mutations can play a role on pear phenotypes, meaning that the study of pear clonal populations can be relevant in genetic studies, mainly when comparing with traditional association studies.

## Introduction

Pears (*Pyrus*) include several fruit tree species cultivated globally, being one of the most economically important fruits with nearly 24 million tonnes produced in 2019 [1]. The *Pyrus* genus is characterized by a high degree of genetic variability, mainly promoted by the outcrossing nature of its species and their interspecific fertility, which together with the recent domestication and dissemination events, becomes a hard puzzle when it comes to establish boundaries and recognize different species. Broadly, the estimates of diversity for *Pyrus* vary between 50 and 80 species and interspecific hybrids [2, 3], although there is a consistent number of around 20 primary, non-hybrid species that are accepted and vastly recognized [4]. Cultivated *Pyrus* species are traditionally divided into two groups based on domestication area and geographic distribution. European pears, *Pyrus communis*, are cultivated mainly in Europe and the U.S. whereas Asian pears like *Pyrus pyrifolia*, *Pyrus*  *bretschneideri*, *Pyrus ussuriensis* and *Pyrus sinkiangensis* are grown in East Asia.

The assessment of *Pyrus* variability has been done in the past using several tools, including molecular markers. These include studies on Asian pears, both wild and cultivated [5–8] as well as European pears [9–12]. In the case of European pear, various studies have been performed to assess the genetic diversity of both cultivated and wild accessions, including studies from Portugal, Spain, Italy, Turkey, Bosnia and Herzegovina, Iran, Tunisia, among others. Despite their relevance on establishing the population structure and phylogeny between accessions, these studies were mainly limited by the resolving power of the few molecular markers used when compared to genome-wide high-density markers such as Single Nucleotide Polymorphisms (SNPs).

In the last decade, several efforts have been made to generate accurate reference genome sequences for pear species. Wu et al. first published in 2013 the draft genome of *P*yrus *bretschneideri* [13], followed by the draft genome of European pear by Chagné and colleagues [14]. Later, improved versions of these genomes were made available [15, 16] as well as the genome sequence of *P. pyrifolia* [17], the genome of the interspecific hybrid “Zhongai 1” [18] and even the genome sequence of wild species *Pyrus betuleafolia* [19]. Altogether, these genomes enable researchers to perform in-depth, high resolution studies at a genome-wide level, which surely will i) contribute to a better understanding of pear genomics, evolution and domestication and ii) play a role in allowing the development of new molecular tools to assist pear breeding. This was surely true for the work developed by Wu and colleagues [20] as, to date, it is the only analysis on *Pyrus* species at scale, using whole genome sequencing technology on 113 globally distributed pear accessions. Their work clarified the processes of divergence and domestication of both Asian and European pears, and it represents the most exhaustive piece of work on the evolutionary history of pears. Concerning novel tools for breeding, reference genomes have also allowed the development of high resolution genetic maps mainly based on SNP markers and quantitative trait loci (QTL) analysis [21, 22], genome-wide association studies (GWAS) [23–25] or even genomic selection (GS) [26].

The studies on pear genetics have been mainly focused on inter- or intraspecific variability, probably due to the inherited difficulty in understanding the evolution and domestication within the *Pyrus* genus. However, research on the intra-varietal (or clonal) variability in pear is scarce, despite that many pear varieties have been clonally propagated for centuries. Clonal populations can be of great use for pear genetic studies, mainly because selfing of individuals is not possible, thus F^2^ populations are never available for such studies. Also, the generation of genetic populations in pear is a longstanding process, with large intergeneration periods, whereas the identification of bud mutants in pear orchards and their genomic analysis is fast. Although not all traits can be studied using clonal populations, simply because there are not enough bud mutants covering all possible traits, this limitation is also true for bi-parental populations that usually only segregate for a specific group of traits as well.

A bud mutant or bud sport is characterized by a somatic mutation that occurs in an adult plant, giving rise to a different phenotype. These mutants are often propagated into clonal progenies, thus retaining the new characteristic and keeping all the remaining plant traits. In fact, several pear varieties grown nowadays are bud sports of another variety, like “Red bartlett” or “Red d’Anjou” that were originated from “Bartlett” (*syn.* “Williams”) and “Beurré d’Anjou”, respectively. Despite their importance for pear genetic resources and overall pear production, the genomics of pear clonal populations is largely unknown. Genome-wide clonal variation was already studied for other highly heterozygous crops, such as apple “Fuji” [27], “Jonathan” and “Sweet Jonathan” [28] as well as for grapevine “Malbec” [29], “Chardonnay” [30], “Nebbiolo” [31] and “Zinfandel” [32]. These studies report a wide range of variants called between clones regardless of the species, ranging from a few hundred to a few million. The first approach to differentiate clones from a pear variety using SSR markers was performed in the Portuguese pear variety “Rocha” [33].

The origin of “Rocha” variety dates back to the 19^th^ century, when a chance seedling was identified in the farm of Mr Pedro Rocha by its superior fruit quality [34]. Nowadays, approximately 99% of all Portuguese pear production comes from “Rocha”, meaning more than 200.000 t per year, spread over more than 12.000 ha and a regular presence in the Top5 of the biggest European producers. Every single “Rocha” tree, in every pear orchard in Portugal, is a clone from that unique, ancient tree. In the 70’s, a breeding program focusing on “Rocha” clonal selection and directed to yield and virus free plants was initiated, and the existence of high clonal variation in farmer’s orchards was discovered. A collection of clones representing this variability was then established in 1985 at Vieira de Natividade Fruit Research Station (Alcobaça) from the National Institute of Agrarian and Veterinarian Research (INIAV, IP). Initially containing more than 80 unique trees, the collection is maintained nowadays in the field and *in vitro* at the Portuguese Plant Germplasm Bank (BPGV). This collection offers a unique opportunity to study the genomics of pear clonal variability and to understand how pear genomes evolve after centuries of clonal propagation.

This work aims to precisely quantify the genomic differences between “Rocha” clones and to infer, based on the base pair resolution of genomic variants, where these differences occur. Is the distribution of somatic mutations even across the pear genome or are there specific regions that tend to accumulate such mutations? Are these regions shared between “Rocha” clones, or does each clone possess a specific mutational signature? To address these questions, we have performed a whole genome resequencing of eight selected clones of “Rocha”.

## Results

### Sequencing, variant calling and genome-wide polymorphisms distribution

In this work, eight pear accessions belonging to the Portuguese pear germplasm collection, representing eight clones of the commercial pear variety “Rocha” were genome resequenced. Sequencing yielded from 70 M to 110 M reads depending on the sample, which accounted for a theoretical mean genome depth of 27× ranging from 21× in PRT 50 to 33× in PRT 51, respectively (Table S1). After filtering, approximately 2% of the reads for each sample were excluded due to quality thresholds. Clean reads were then mapped to the reference genome and only those reads mapping with a quality of 30 or above were considered. Reduction of reads due to mapping quality filtering ranged from 34% in sample PRT 58 to 47% in PRT 50. In the end, after all filtering steps, mean depth ranged from approximately 11× in PRT 50 to 20× in PRT 51, averaging 16× (Table S1). The genome coverage (i.e. the proportion of genome covered with at least one read) ranged from 77.6% in PRT 50 to 87.73% in PRT 51, averaging 86.1%. All samples presented 70% or more of their genome covered at least 10 times, with the exception of PRT 50 (Table S1).

After variant calling and filtering, 3 087 709 SNP positions and 339 628 INDELs were identified for the 17 chromosomes belonging to the eight “Rocha” clones. All data presented in this manuscript concerned the 17 pear chromosomes only, and did not include unassigned scaffolds and contigs. In detail, the number of SNPs per sample was stable around 3 million polymorphisms (from 2 941 735 to 3 025 490; Table S2A), with the exception of PRT 50 (2422271). Similar trends were found for INDELs, which varied between 316 087 and 327 382 with the exception of PRT 50 (240 993 INDELs). Due to the low coverage of sequencing, 566 515 SNPs and 72 724 INDELs were filtered out for sample PRT 50 (18.95% and 23.28% of total SNPs and INDELs called for this sample, respectively) whereas for the remaining samples the percentage of discarded SNPs never exceeded 3.87% and discarded INDELs never exceeded 4.62%. Variants validation was performed by Sanger sequencing of PCR amplified locus containing nine SNPs and four indels from “Rocha” clone PRT 52. Three primer pairs did not amplify any detectable fragment and were excluded. One primer pair amplified several fragments and was also excluded. For the remaining nine loci variants, seven (78%) presented the expected genotype as called *in silico*, including five SNPs and two INDELs (Table S7).

Overall, the distribution of missing data per chromosome across the SNP dataset (i.e. the amount of SNP positions that had an unknown genotype for four samples or less) is shown on Table S3A. The great majority of SNP positions had a valid genotype in all eight “Rocha” clones (2 385 442 out of 3 087 709; 77.25%)(Figure S3). Considering all SNPs with valid genotype in at least seven samples, available positions increase to 2 864 498 or 92.77% of all SNPs. The SNP frequency between “Rocha” and the reference genome “Bartlett” was found to be approximately 7.3 SNPs every Kbp (Table S2A). Similar results were obtained for INDELs (Table S2B). The great majority of INDEL positions had a valid genotype for all eight samples (248 667 positions out of 339 628; 73.21%) and the estimated variability between “Rocha” and “Bartlett” genomes was around 0.79 INDELs per Kbp (Table S2B).

**Figure 1:**
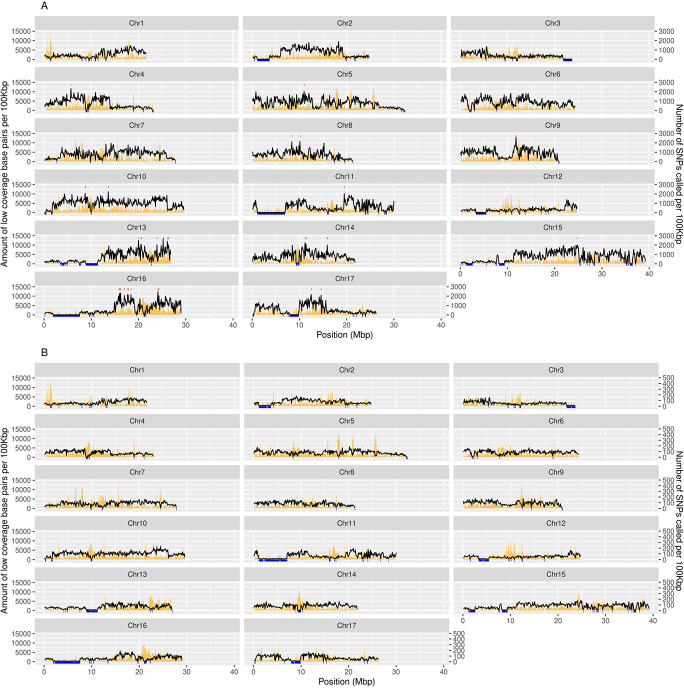
Overall “Rocha” SNPs (A) and INDELs (B) distribution across all 17 pear chromosomes. Blue bars at the bottom represent regions with very low variability. Top red bars represent regions with very high variability. Orange background represents the amount of low coverage base pairs (<5×) along the chromosomes. Black line represents the number of SNPs or INDELs called.

The above mentioned rate of SNPs between “Rocha” and “Bartlett” genomes was uneven in the genome, depending on the chromosome considered, but not correlated with chromosome size (ρ = 0.303, α > 0.1). Furthermore, within each chromosome there was also great variability regarding SNP density, with both high and low SNP rate regions (Figure 1A). A relative abundance of regions with low – almost null – variability (i.e. 1 SNP/Kbp or rarer) between “Rocha” and “Bartlett” genomes was found, across all chromosomes. The size of such low variability regions, however, greatly varied between chromosomes, and was as extensive as 6–7 Mbp (Chr11 and Chr16; Figure 1A). In total, these very low variability regions accounted for 41.1 Mbp, which is roughly 1/10 of the combined size of the 17 chromosomes available for the “Bartlett” reference genome. In contrast, highly variable regions (1 SNP every 50 bp or more frequent) were also widely detected, although the extension of such regions was short. Similarly, the distribution of INDELs was uneven across the genome, with high and low variability regions (more than 2 INDEL/kbp or less than 0.2 INDEL/kbp, respectively) also occurring for these type of variants (Figure 1B). The variation in the abundance of both SNPs and INDELs across the genome did not correlate with the availability of genome coverage for variant calling (Figure 1A-B, orange background).

### “Rocha” genome evolution and the relatedness among “Rocha” clones

The nucleotide diversity for the population of eight “Rocha” clones was estimated using a SNP dataset, without missing data, with 233 134 polymorphic SNPs. Nucleotide diversity (π/bp^−3^) ranged from 0.029 in chromosome 12 to 0.127 in chromosome 6 and chromosome 10 (Table 2). Similar results were found for the Watterson estimator (θ_w_*/*bp^−3^), with the lowest genetic variability being 0.041 for chromosome 12 and the highest being 0.157 for chromosome 6. The distribution of the nucleotide diversity using 50 Kbp non overlapping sliding windows was also investigated (Figure S2A-B). Chromosome 12 clearly presented a low diversity in all of its length, similar to chromosome 3. On the other hand, the most diverse chromosome 6 also had a high diversity throughout, but the highest peaks of nucleotide diversity were found in chromosomes 13, 14 and 15.

**Table 1:** The origin, name year of collection and unique traits of the eight “Rocha” clones studied in this work

**Accession number**	**Taxon**	**Name**	**Origin**	**Year of collection**	**Unique traits**	**Maintenance Site**
PRT 11	*Pyrus Communis*	Carapinheira	Porto	2013	*na*	ENFVN
PRT 50	*Pyrus Communis*	Rocha Cl 1	Campelos, Torres Vedras	1985	Irregular fruit shape	ENFVN
PRT 51	*Pyrus Communis*	Rocha Cl 2	Quinta da Barrada, Alcobaça	1985	Less alternate bearing	ENFVN
PRT 52	*Pyrus Communis*	Rocha Cl 3	Alcobaça	1985	Very irregular fruit shape and size; Larger leaves; smaller flower organs	ENFVN
PRT 53	*Pyrus Communis*	Rocha Cl 4a	Quinta da Roda, Alcobaça	1985	Larger fruits	ENFVN
PRT 55	*Pyrus Communis*	Rocha Cl 4b	Quinta da Roda, Alcobaça	1985	Higher fruit production	ENFVN
PRT 56	*Pyrus Communis*	Rocha Cl 5	Várzea de Cós, Alcobaça	1985	Higher fruit production	ENFVN
PRT 57	*Pyrus Communis*	Rocha Cl 6	Várzea de Cós, Alcobaça	1985	Higher russeting	ENFVN
PRT 58	*Pyrus Communis*	Rocha Cl 7	São Martinho do Porto, Alcobaça	1985	Longer fruits; higher russeting	ENFVN

**Table 2:** Covered bases, estimates of nucleotide diversity (π and the Watterson estimator θ_w_) and Tajimas’ D for each chromosome or genomic region. ^*^Covered bases where all eight clones had at least 5× coverage each

**Genomic region**	Covered bases^*^	**Polymorphic sites (SNPs)**	**Nucleotide diversity (π/bp** ^**−3**^**)**	**Nucleotide diversity (θ**_**w**_ **/bp** ^**−3**^**)**	**Tajimas’ D**
Genome	332 524 351	233 134	0.070	0.114	−1.096
Intergenic	258 325 090	215 341	0.087	0.145	−1.072
Intron	38 699 271	11 185	0.016	0.006	−1.269
Exon	35 499 990	6608	0.009	0.004	−1.079
Chr1	16 250 767	10 756	0.059	0.078	−1.336
Chr2	18 784 484	13 887	0.090	0.114	−1.160
Chr3	18 524 145	9648	0.037	0.050	−1.449
Chr4	16 283 254	13 426	0.095	0.117	−1.028
Chr5	22 858 130	17 141	0.094	0.115	−1.019
Chr6	17 423 142	16 816	0.127	0.157	−1.046
Chr7	19 565 425	18 224	0.114	0.142	−1.060
Chr8	16 028 562	13 008	0.099	0.123	−1.069
Chr9	14 688 684	10 908	0.106	0.126	−0.862
Chr10	21 874 763	18 083	0.127	0.153	−0.937
Chr11	23 432 135	11 824	0.056	0.068	−0.943
Chr12	19 210 719	9878	0.029	0.041	−1.669
Chr13	19 668 738	12 358	0.094	0.111	−0.851
Chr14	16 061 159	13 398	0.110	0.135	−0.998
Chr15	30 725 940	19 128	0.092	0.113	−0.988
Chr16	21 200 686	11 510	0.083	0.096	−0.757
Chr17	19 943 618	13 141	0.072	0.091	−1.184

In order to understand the evolution of the “Rocha” genome in terms of the randomness of its mutations, and to test whether or not the frequency of polymorphisms in this population matched the history and origin of “Rocha”, the Tajima’s D was also calculated for all chromosomes (Table 2).

**Figure 2:**
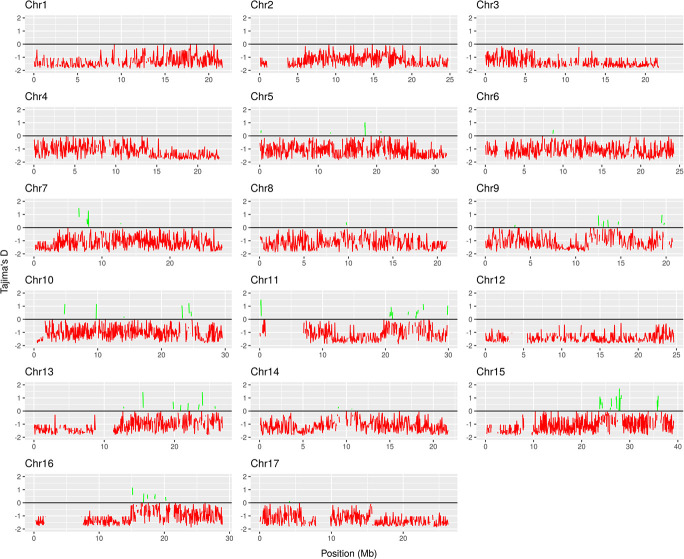
The distribution of tajima’s D values, for the “Rocha” population, in 50 Kbp windows for all 17 chromosomes of pear. Red line represents windows with negative Tajima’s D values. Green line represents positive Tajima’s D values.

The lowest values for Tajima’s D were found in chromosome 12 (−1.669) and the highest in chromosome 16 (−0.757). Using sliding windows of 50 Kbp, several regions with positive values of tajima’s D were identified, some of them significantly deviating from the chromosome average (Figure 2). Two regions in particular were isolated, for chromosomes 15 and 17, that presented a positive tajima’s D value higher than 1.5 (Table 3). For chromosome 15, there were four genes annotated in “Bartlett” genome for the selected region (27.80–27.85 Mbp). In chromosome 17, there were three genes annotated in the positive tajima’s D region (4.55–4.60 Mbp). The nucleotide diversity within these regions was also very high, mainly for chromosome 15, with values of π/bp^−3^ and θ_w_*/*bp^−3^ of 4.178 and 3.085, respectively (Table 3). The genes that were annotated in these two regions had GO assignments of ATP binding, protein binding, translation initiation factor activity, chromatin remodeling and (1- > 3)-beta-D-glucan biosynthetic process (Table 3).

**Table 3:** The two regions of 50 kbp with Tajima’s D values higher than 1,5 in the “Rocha” clonal population, the nucleotide diversity of these regions, the genes annotated and their Gene Ontology (GO)

**Chromosome**	**Region**	**π bp** ^**−3**^	**θ**_**w**_ **bp**^**−3**^	**Tajimas’D (upstream)**	**Tajimas’D in the region**	**Tajimas’D (downstream)**	**Genes annotated in the region**	**GO**
15	27 800 000 – 27 850 000	4.178	3.085	0.283	1.713	−0.095	pycom15g30380	(1- > 3)-beta-D-glucan biosynthetic process; 1,3-beta-D-glucan synthase activity; 1,3-beta-D-glucan synthase complex; membrane
							pycom15g30390	chromatin remodeling; DNA binding; DNA repair; histone H2A acetylation; histone H4 acetylation; NuA4 histone acetyltransferase complex
							pycom15g30400	—
							pycom15g30410	protein binding
17	4 550 000 – 4 600 000	1.607	1.157	−1.31	1.601	−0.755	pycom17g06360	cytoplasm; eukaryotic translation initiation factor 3 complex; translation initiation factor activity
							pycom17g06370	ATP binding; protein kinase activity; protein phosphorylation
							pycom17g06380	ATP binding; protein kinase activity; protein phosphorylation

**Figure 3:**
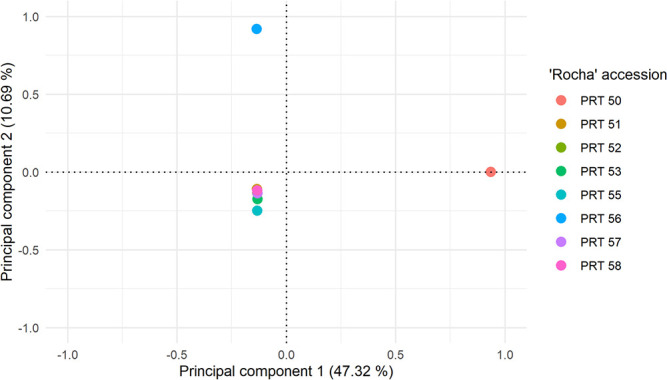
Principal component analysis (PCA) using genome-wide SNP data generated in this study for the eight “Rocha” samples. Only polymorphic SNPs with valid genotypes across all samples were used (233 134 SNPs).

To further understand the genetic relationship among clones we performed a Principal Component Analysis (PCA) of the SNP variants (Figure 3). PCA clearly separated “Rocha” clones into three main areas. PRT 50 was undoubtedly separated from the rest of the clones based solely on component one, which explained 47.32% of overall variation. PRT 56 was also separated from all other clones, and its positioning was also mainly explained by the variation of a single component, in this case component two (10.69%). All the remaining clones grouped together in a single cluster.

A Kinship analysis was also performed in order to quantify the relationships between “Rocha” clones. SNP data from a local cultivar “Carapinheira” (Accession: PRT 11), without any known relationship with “Rocha” variety, was also included in the analysis (Figure S4). The values of relatedness ranged from 0.0 (not related) in the case of all clones vs “Carapinheira” to 0.49 between all clones with the exception of PRT 50 (highly related; 0.5 is selfing). PRT 50 was the least related with the remaining clones, presenting a relatedness value of 0.45 for all clones.

### The clonal variability in “Rocha” is uneven and presents both low and high mutation regions

From the 233 134 polymorphic SNP variants among clones, 183 407 of them (78.67%) were exclusive for a given clone, meaning that the majority of intra-varietal genomic variation found in “Rocha” is unique to a given accession and not shared. The remaining 49 727 variants (21.33%) were shared by two or more clones. These values were however misleading, since PRT 50 was the main contributor for the overall picture of intra-varietal variability. In detail, PRT 50 alone had 127 563 SNP variants that were exclusive to its genome. In contrast, the remaining 7 clones had an average of 7071 unique SNPs, ranging from 4848 in PRT 51 to 12 955 in PRT 56. The overall frequency of unique SNPs for each clone varied greatly considering 1 Mbp sliding windows, with an average of 282 SNPs/Mbp for PRT 50, 28 SNPs/Mbp for PRT 56 and between 10 and 20 SNPs/Mbp for the remaining clones. Taken together, the macro distribution of unique SNPs had the same tendency in all clones. When looking for the Kbp resolution however, the differences among clones became evident, and all variability was found to be accumulated in very narrow locations. The coverage of sequencing was comparable between clones and the regions without unique SNPs did not correlate with low coverage regions. It also became evident that the greater rate of mutation found for PRT 50 came from a wider dispersion of mutations across the genome, rather than an accumulation in small regions (i.e. higher accumulation in the same Kbp).

To better understand the nature of the regions that accumulated the most mutations, we searched for DNA motifs that could be overrepresented in fragments with high mutation load. In total, 72 DNA motifs were found significantly enriched in these regions, when compared with regions of the same size (1 Kbp) without any mutation recorded. The five most significant DNA motifs for each class of region are presented in Figure S5. All significant motifs obtained were compared with available *A. thaliana* databases of known motifs. Results from this comparison are detailed in Table S4. Fourteen out of the total 72 DNA motifs discovered in regions of high or very high mutation had a match, the majority of which were known transcription factors.

### The annotation of variant effects among “Rocha” clones highlights the theoretical disruption of key genes in pear

**Figure 4:**
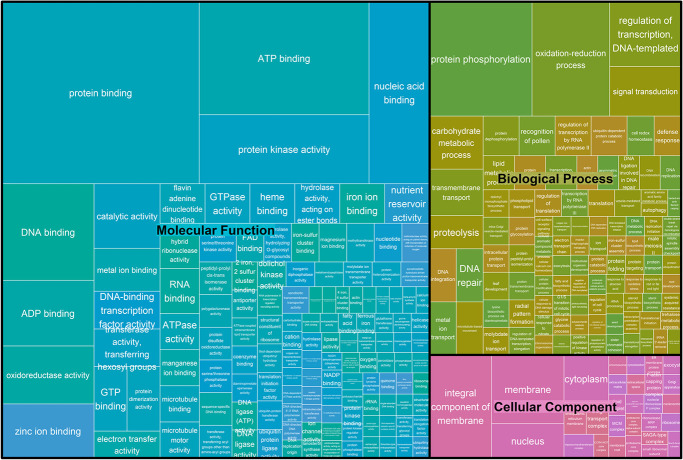
GO assignments of genes affected by HIGH impact SNP variants in “Rocha” clones. The size of rectangles is directly correlated with the number of occurrences of a given GO.

The putative impact of variants detected in “Rocha” clones was estimated using only polymorphic variants (233 134 SNPs and 43 954 INDELs). After annotation, those classified as having HIGH impact were isolated and inspected manually. In total, 216 SNP and 557 INDEL variants were classified in this category, which corresponds to 0.09% and 1.27% of all polymorphic variants, respectively. In addition, 168 SNPs and 425 INDELs were exclusive to a given clone and not shared. Within the SNP variants, the most common type of impact was “stop gained”, accounting for 50.1% of all types of HIGH impacts annotated (Table S5). In the case of INDELs, the most common was “Frameshift variant”, accounting for 89% of all types of impact. The genes that were impacted by these variants had a diverse distribution regarding their gene ontology annotations (Figure 4), with most common ontologies being protein binding, ATP binding, Protein phosphorylation, protein kinase activity and nucleic acid binding.

### The majority of transposable element insertions in “Rocha” clones are unique

When compared to the “Bartlett” reference genome, PoPoolationTE2 and Retroseq yielded 1198 and 7518 new TEI in “Rocha” clones, respectively. Surprisingly, these TEI were mainly exclusive to one given clone rather than shared between several or all clones (Table S6). In detail, following Retroseq results, 46.67% of all new TEI (3509) were exclusive to a given clone, 23.48% (1765) were shared between two clones and only 1.3% (98 TEI) were shared between all “Rocha” clones. Class I LTR retrotransposons were the most common among all new TEI detected (4075 out of 7518), followed by Terminal Inverted Repeat (TIR) and Miniature Inverted-repeat Transposable Elements (MITEs) class II transposons. For PoPoolationTE2 analysis the results were similar: 53.1% were exclusive to one clone, 21.37% were shared between two clones and only 0.58% (7 TEI) were shared between all clones. DNA transposons were the most common, mainly TIR and MITEs, followed by LTR and SINE retrotransposons (Table S6). TEI unique to one given clone were found to be inversely correlated with the amount of unique SNPs for that same clone (ρ = −0.71, α < 0.05 - PoPoolationTE2; ρ = −0.93, α < 0.005 - Retroseq). In terms of location and distribution, for PoPoolationTE2 TEI accumulated mainly in three genomic regions, regardless of the clone where they were detected. These regions included the distal end of chromosomes five, eleven and fifteen, comprising a total of 20–25 Mbp (Figure S6B). In contrast, the distribution of non-reference TEI discovered with Retroseq was rather uniform, matching the overall SNP and INDEL density (Figure S6A vs Figure 1A/B).

## Discussion

### The intraspecific variability in *P. communis*

Pears comprise a very rich genus with both wild and cultivated species, with different levels of ploidy and several hybrid species. Also, many pear traits are genetically complex, and others are still to be determined from a genetics perspective. This clearly indicates that genetic and genomic studies are much needed for *Pyrus*, to clarify the relationship among species, to study and compare pear genomes and their evolution, and to help develop new molecular tools to assist the breeding of key pear traits.

In this study, 3 087 709 SNPs and 339 628 INDELs were called for the 17 chromosomes belonging to “Rocha” clones. This represents one measure of intraspecific variability of cultivated European pear and translates into 7.3 SNPs and 0.79 INDELs/Kbp. The only pear resequencing publication to date from Wu and colleagues [20] analysed 50 European pear accessions and reported a larger number of SNPs than those found for “Rocha” (6 945 796 or 13.2 SNP/Kbp). However, only 25 out of those accessions were cultivated European pear (*Pyrus communis*), and the SNP number on those 25 samples was much lower, around 4 220 232 SNPs or 8.02 SNPs/Kbp, which is almost in line with this study, despite the fact that the reference genome used there was the Asian pear genome, *Pyrus bretschneideri* “DangshanSuli”. Thus, Wu’s result of 8.02 SNP/Kbp reflects the overall variability between *P. communis* samples and *Pyrus bretschneideri* genome, which translates to *Pyrus* interspecific variability. Another publication by Montanari et al [56], on the development of a 70 K pear SNP array, reported 3 809 750 SNPs detected for the 19 samples of the “communis group”, which includes both wild and cultivated *P. communis*. Montanari’s results on the variability of European pear translate into 7.24 SNPs/Kbp, which is in total agreement with the results presented here, even considering that a previous version of “Bartlett” reference genome (V1.0) was used for read mapping, and that their group of 19 samples included some wild European pear species.

Pear fertility is controlled by a gametophytic self-incompatibility system, a mechanism that promotes outcrossing and prevents selfing or crosses between common genome backgrounds, based on the alleles of the S-RNase gene. As a consequence, pears display highly heterozygous genomes. Nevertheless, some genomic regions present identity-by-descent (IBD), characterized by very low variability even when comparing different *Pyrus* species [20]. In this work, roughly 10% of “Rocha” genome was discovered with a SNP rate lower than 1 SNP/Kbp over “Bartlett”, suggesting the presence of large IBD regions. Such regions are expected between modern pear varieties due to the reduced number of founder lines used in breeding programs in the past. Indeed, “Rocha” and “Bartlett” are both supposed to be direct descendants of “Whyte Doyenne” [57], meaning they should be half siblings sharing ~25% of their genome. However, the results from this work suggest that these varieties are third degree relatives (~12.5% genome shared) rather than second degree relatives. This may be the first relatedness coefficient estimation for “Rocha” and “Bartlett” pair, which may contribute to solve the unresolved pedigree of “Rocha” cultivar.

### The clonal variability of “Rocha” pear

For perennial crops, the understanding on how a given variety accumulates mutations over time is of key interest, given that these crops are mainly propagated clonally to maintain their traits, thus carrying their somatic mutations forward. In this study, from the global dataset of “Rocha” vs “Bartlett” SNP variants called in all samples, only 233 134 were polymorphic (i.e. at least one clone had a different genotype from the remaining clones). In other words, the genomic variation at the “Rocha” clonal population level (intra-varietal variation) is estimated at 1/10 (233 134 vs 2 385 442) of the genomic variation of “Rocha” vs “Bartlett” (intraspecific variability). This measure is higher than apple “Fuji” clonal variability [27], where the number of SNP variants between “Fuji” and the apple reference genome “Golden delicious” was 1 744 187 SNPs, whereas between “Fuji” and any of its bud mutants was approximately 51 000 SNPs, representing 1/34 of the intraspecific variability. However, the decreased variability found in apple “Fuji” clones when compared with this study in pear may be explained by the short period since the inception of the “Fuji” variety, which was only released to the market in the 1950’s and the “Fuji” clones studied by [27] were isolated not before the 1980’s. Another study on apple bud mutants studied the genome-wide differences between “Jonathan” and “Sweet Jonathan” and found as much as 4 198 955 SNPs between those two clones [28], representing 84 times the intra-varietal variation on apple “Fuji” from [27]. Grapevine is another outcrossing crop species that has been studied for clonal variability. There are studies reporting as low as 941 intra-varietal SNPs for “Malbec” [29], a few thousand SNPs for “Chardonnay” [30] and “Nebbiolo” [31] and more than 630 000 SNPs for “Zinfandel” clones [32]. This comes to show that the study of clonal diversity is a challenging task and results are somewhat inconsistent, either likely due to methodology such as the differences in sequence coverage, quality filtering and overall pipeline design or due to the propagation record and the history of the plant material.

In terms of overall nucleotide diversity, “Rocha” clonal population presented a genome-wide value of 0.070 π/bp^−3^. For reference, this compares with 3.53 π/bp^−3^ for a population of 25 *P. communis* cultivars [20]. Tajima’s D values were highly negative for every chromosome and for the overall genome (−1.096), and more negative than at intraspecific level, as expected (−0.78 [20];). Nevertheless, when calculating both nucleotide diversity and Tajima’s values over 50Kb sliding windows, highly variable regions among clones and high positive Tajima’s D values were found. Two examples of such regions are presented in Table 3 where several key genes are annotated in “Bartlett” genome, including callose synthase, two Malectin Receptor-Like Kinase (RLK) genes and two SWR1-complex protein 4-like isoforms, among others. In *A. thaliana*, Callose synthase is required for the formation of cell walls during pollen development and may be involved in callose synthesis during pollen tube growth. During plant development, callose is also found as a transitory component of the cell plate in dividing cells and it is found as a structural component of plasmodesmatal canals [58, 59]. On the other hand, RLK Kinases have been related with immunity responses and overall resistance to pathogens in several plant species [60, 61]. However, RLK Kinases from the FERONIA subfamily (FER and ANX1/2) also play a key role during communication between pollen tube and synergids, making these genes required for normal fertilization in plants [62, 63]. In the *Pyrus* genus, the RLK family of proteins were studied in-depth by [64], with 26 genes in total being identified in *Pyrus bretchneideri* genome. From those 26, five genes were classified as belonging to the FER and ANX1/2 subfamily (PbrCrRLK1L3, PbrCrRLK1L5, PbrCrRLK1L8, PbrCrRLK1L11, and PbrCrRLK1L26). Kou and colleagues (2017) showed that the expression of PbrCrRLK1L3 and PbrCrRLK1L26 is higher during pollen tube growth when compared with other RLK genes from other subfamilies, and they proved that pollen tube elongation is halted when the expression of these two genes is inhibited. The two RLK genes in the highly variable region presented here (pycom17g06370 and pycom17g06380) have their best BLAST hits with *Pyrus bretchneideri* FER family genes, suggesting that they could also be required for pollen tube elongation in *P. communis*. On the other hand, pycom17g06370 and pycom17g06380 also showed great similarity with *Malus domestica* RLK FERONIA genes, which were recently proven to be involved in the regulation of apple fruit ripening [65]. Regarding SWR1-complex protein 4 (SWC4) genes, these are part SWR1-complex which mediates the ATP-dependent exchange of histone H2A for the H2A variant HZT1, leading to transcriptional regulation of selected genes by chromatin remodeling in *Arabidopsis* (UniProtKB:Q8VZL6). *Arabidopsis* artificial miRNA knock-down SWC4 (*Ateaf1*) lines showed decreased levels of H4K5 acetylation in the promoter regions of major flowering regulator genes and showed early flowering [66]. Altogether, in this work, several genes annotated in the two highly variable 50Kbp regions have orthologs with key functions described for plant fertility and flower development in other plant species. This suggests that balancing selection of fertility-related genes could be occurring in the “Rocha” clonal population. However, since all clones were initially isolated and selected based on the presence of distinctive traits, these highly variable regions are most likely a consequence of human selection. Future research with a larger sample dataset can corroborate these results and allow for a better understanding of these events in the collection.

The relatedness among “Rocha” clones was explored through PCA and Kinship analysis. These approaches revealed that PRT 50 is the least related with the remaining clones, followed by PRT 56. The kinship coefficient is equal to 0.49 for every pair of clones, with the exception of PRT 50 (0.45). These results are partially in agreement with the results by [33] using SSR markers in the same biological material and suggest the origin and the history of propagation of clones as the most likely explanation for their divergence. This is mainly true for PRT 50, since the orchard where it was identified ~40 years ago was located in the region of *Torres Vedras*, closer to the location of the original “Rocha” tree. All the remaining clones were identified in orchards from the region of *Alcobaça*, with little geographical distancing between them (Figure S1). This correlation between genetic and geographical distancing could reflect the sharing of grafting material between farmers in the 60’s or 70′, or even the adaptation of clones to differing conditions, although this cannot be confirmed.

The relatedness between pear clones found in this work can be compared with other outcrossing crops like grapevine. Vondras et al [32] found that the 15 “Zinfandel” clones had kinship coefficients between 0.42 and 0.45, which suggests that grapevine clones tend to be less related than European pear ones. However, “Zinfandel” was confirmed to be the ancient Croatian variety “Tribidrag”, for which there are records of cultivation in Croatia for as early as the 15^th^ century [67], meaning that its propagation history is at least 500 years old. Thus, the lower relatedness of “Zinfandel” clones is most likely a consequence of its extended clonal propagation, which certainly led to a greater accumulation of somatic mutations.

Clone-specific mutations (i.e. SNPs that are present in one clone and absent in the other clones) were not distributed equally in the genome. At the Mbp level, all clones presented the same tendency, but when searching at the Kbp resolution, some Kbp windows were targeted for SNP accumulation, but those windows were usually not shared between clones. Several DNA motifs were overrepresented at these locations, 14 of which had a match with available databases. The majority of these matches were associated with families of transcription factors that are known to play important roles regulating gene expression in plants, namely Basic leucine zipper (bZIP), NAC, basic helix–loop–helix (bHLH) and WRKY [68]. This result shows that, despite the fact that somatic mutations are likely to be present throughout the genome, in European pear “Rocha” they can also occur close to transcription factors, thus reinforcing their role in the emergence of new pear phenotypes. This was corroborated by the annotation of variants, which could be directly impacting the coding sequence of genes. In this work, 216 SNPs and 557 INDELs with potential to cause protein truncation or loss of function were annotated. From those, 168 SNPs and 425 INDELs were unique to a given clone. This set of polymorphisms can be responsible for some of the unique phenotypes that can be found in the “Rocha” clonal collection, as hinted by the ontology of the gene affected, and they can contribute to the study of European pear genetics by uncovering the causal genes of key phenotype.

### Each “Rocha” clone has a unique signature of transposable elements

In this work several thousand new TE insertions were found in “Rocha”, but surprisingly those insertions were hardly shared among clones. In fact, unique TE insertions (those only detected in one single “Rocha” clone) accounted for approximately half of all insertions, and only ~1% were shared between all eight clones. This is unexpected and is somewhat in disagreement with the history of “Rocha”, since theoretically all individuals were propagated from a single ancient pear tree, meaning that most genetic variants that are exclusive to “Rocha” and not present in “Bartlett” reference genome should be shared between clones (which is true for SNPs and INDELs).

One possible explanation for these results is that the genomic location of TE in European pear is mostly common between cultivars, hence the majority of “Rocha” TEs are also present in “Bartlett”, thus excluding the most “shared” TE from the analysis, since only non-reference insertions were considered (new TE insertions). Another factor that must be considered to understand this result is the origin of “Rocha” cultivar. Historic records mention a single tree, nearly 200 years ago, as the origin of all “Rocha” that exists nowadays [34]. However, the uniqueness of TE location in this clonal collection suggests otherwise. If “Rocha” would have multiple origins or starting materials for propagation in its inception as a cultivar, it could explain the profile of TE found in the clonal population of this work. However, even considering multiple origins, a great degree of TE sharing would be expected that could reflect a common genomic background vs “Bartlett”. Finally, it is also worth mentioning that the quality of sequencing data can play a role in the results, mainly because the coverage and quality of sequencing varies depending not only on the region of the genome but also from sample to sample. In other words, a given TE in a given genomic location is only shared by *N* clones if there is sufficient sequencing on that specific region, in all *N* clones, to confidently call that TE.

Despite being unexpected, the uniqueness of new TE insertions in clonal populations is described in the literature for other perennial crops [32]. Future work will help to understand what drives the movement of TE in constantly propagated perennial crops, and what are the implications for clonal integrity and genome stability.

## Conclusions

The genomic studies of pear species are still scarce. The understanding of the pear genome and its features, as well as the study of the genetics behind many key traits in pear are crucial to assist the breeding of new genetic combinations. This will be very important for pear to adapt to climate changes and to meet other challenges that producers are already being faced with, including a transition into a greener agriculture. Clonal populations of pear can play a role in the understanding of pear genomics, uncomplicating its genome due to high similarity between individuals, thus overcoming the typical complexity of an outcrossing species. In this work we have identified several variants between “Rocha” clones occurring inside coding regions of genes that can be responsible for some of the unique phenotypes presented by these clones. In the future, the in-depth study of somatic variants may unveil the genetics behind several pear traits that are still to be determined to date, opening new opportunities for Marker Assisted Selection (MAS) and Breeding.

## Materials and methods

### Plant material

Eight pear accessions from the Portuguese pear germplasm collection, representing eight clones of the commercial variety “Rocha” were used in this study. These accessions were identified in several orchards in the west region of central Portugal in the late 70’s and early 80’s (Figure S1), and were grafted in 1985 at the Vieira de Natividade Fruit Research Station (Alcobaça, Portugal). Additionally, a Portuguese local variety “Carapinheira” was also used in this study, but mainly for comparison purposes or as an outgroup. All plant materials are registered in the Portuguese node of the GRIN-Global database (http://bpgv.iniav.pt/gringlobal/) under the accession numbers PRT 11, PRT 50, PRT 51, PRT 52, PRT 53, PRT 55, PRT 56, PRT 57, PRT 58 (Table 1).

### DNA extraction and sequencing

Young leaves were collected from year-grown twigs at four positions in each tree, representing the four cardinal points. DNA was isolated using the innuPREP Plant DNA Kit (Analytik Jena AG, Berlin, Germany), according to the manufacturer’s protocol.

After extraction, genomic DNA was checked for quality parameters according to sequencing provider instructions, namely for integrity (using agarose gel electrophoresis), minimum concentration of 20 ng/μL (Qubit®) and purity (absorbance at 260, 280 and 230 nm using NanoDrop ND2000 spectrophotometer (Thermo Scientific, Massachusetts, MA, USA)). Samples were shipped to Novogene Europe (Cambridge, UK) for short-insert paired-end library preparation (2x150bp) followed by whole genome sequencing (WGS) in the Illumina Novaseq platform.

### Preprocessing, mapping and variant calling

Raw reads were filtered following very restrictive quality criteria using cutadapt v.1.18 [35]. Reads containing adapter sequences were trimmed and adapters were removed. Read ends with quality below 30 were also trimmed and low quality read extremes were excluded. Trimmed reads with less than 50 bp were discarded. All reads containing unknown nucleotides (“N”) in its sequence were also excluded from the dataset. Clean reads were then checked for their quality using fastqc v0.11.8 [36] and were mapped against *P. communis* Bartlett DH Genome v2.0 [15] using bwa-mem with default parameters [37]. The resulting alignment was then quality filtered using samtools v.1.9 [38], excluding all reads with mapping quality below 30. Additionally, duplicate reads were also excluded from the alignment dataset using Picardtools Markduplicates v.2.18.14 [39]. The final filtered alignment was then manually inspected for each sample using qualimap bamqc v.2.2.1 [40]$\square $.

All variant calling and filtering were performed using Genome Analysis Toolkit (GATK) v4.0.11.0 [41] following all best practices and recommendations. In detail, SNPs and small insertions/deletions (INDELs) were called for each sample separately using HaplotypeCaller with default parameters, emitting reference confidence scores in genomic variant call format (-ERC GVCF), as recommended by GATK documentation. Individual raw gvcf files were then imported into a GenomicsDB workspace using GenomicsDBImport. The joint genotyping of all samples into a single raw vcf file was then accomplished by using GenotypeGVCFs. Variants were filtered with GATK’s VariantFiltration excluding all sites with a depth of coverage (DP) below 5 or quality by depth (QD) below 20. In addition, Bcftools v1.9 [42] was employed to select only biallelic sites and to filter out all SNP variants that were within 15 bp of any INDEL. In the case of INDELs, a minimum distance of 50 bp (−G 50) between a given pair of INDELs was required. Furthermore, all INDELs with more than 100 bp in length were discarded (−e “ILEN > 100”). A final dataset for quantification of the “Rocha” vs “Bartlett” variability was created by excluding all variants with missing data above 50% (i.e. any SNP/INDEL with missing genotype in more than four “Rocha” clones), using vcftools v0.1.16 [43]. A second dataset without missing data, and considering the polymorphic variants only (see section “Intra-varietal variation”), was created for downstream analysis.

### Validation of variants

Primers were designed for nine SNPs and four INDELs with Primer3Plus [44] around the target sites of sample PRT 52, to allow the amplification of a ~ 300 bp DNA fragment. Primers were blasted against pear reference genome to confirm their location (Table S7). After amplification, fragments were sequenced in-house using an ABi Genetic Analyser 3500 apparatus. The validation of variants was performed by comparing the sequence obtained through Sanger sequencing and the expected genotype called *in silico*.

### Nucleotide diversity and population genomics

Nucleotide diversity (*π*) and the Watterson estimator (*θ_w_*) were calculated using R software [45] through the software package “PopGenome” [46] for each one of the 17 chromosomes of “Rocha” clones, both globally and also following a non-overlapping sliding window approach of 50kbp. Similarly, Tajima’s D was also calculated at the chromosome level as well as in 50kbp windows using the PopGenome package. The kinship coefficient between “Rocha” clones was calculated with KING software [47]. The resulting KING output was converted to a matrix using the function kingToMatrix from “GENESIS” package [48] in R. Principal component analysis (PCA) for “Rocha” clones was calculated using PLINK v1.9 [49]. The top two principal components were plotted in R using the “ggplot2” package [50].

### Sequence signatures of high mutation regions

MEME suite package [51] was used to discover DNA motifs that could have greater occurrence in high mutational regions compared with regions depleted of variation. The creation of an input sequence file for the STREME program was as follows: first, all windows with 10 Kbp in length without any mutation recorded for any clone were isolated. Then, for all those regions, only their middle portion was extracted, from coordinates 4250 to 5750, and the dataset of 1500 bp control (negative) sequences in fasta format was built. Second, all 1 Kbp windows where a given clone has accumulated more than five mutations were isolated. A flanking sequence of 250 bp each side of the 1 Kbp window was also considered, for a total length of 1500 bp fragments. From the fragments containing mutations, two datasets were built: one considering all sequences (high mutation dataset – more than 5 mutations) and other containing only sequences with higher incidence of mutations (very high mutation dataset – more than 10 mutations). These two datasets of primary (positive) sequences were then used, together with the control dataset, to calculate the significance of motifs found in the positive set compared with their occurrence in the negative set.

DNA motifs found to be significantly represented in the positive sequences were then compared with known motifs previously described in the literature using Tomtom tool from MEME suite available at (https://meme-suite.org/meme/tools/tomtom). In detail, all significant motifs were compared with two *Arabidopsis thaliana* databases made available in the web platform, and all matches with e-value below 0.1 were considered valid motif matches.

### Intra-varietal variation

Since there is no “Rocha” reference genome available for variant calling, the intra-varietal variation could not be directly estimated from “Rocha” vs “Bartlett”. However, an intra-varietal variation was considered when a given variant was polymorphic in the population (i.e. when at least one clone had a different genotype call when compared with the remaining clones). Polymorphic positions were also used to isolate unique genotypes as those positions were one clone was different from all the remaining seven clones (i.e. one clone “0/1” vs all the remaining clones “1/1”).

### Estimation of variant effects

The estimation of variant effects was performed using SnpEff software v4.3t [52]. A local database for *P. communis* Bartlett DH Genome v2.0 annotation was built following SnpEff instructions, using the annotation file available at the Genome Database for Rosaceae (GDR) (ftp://ftp.bioinfo.wsu.edu/species/Pyrus_communis/Pcommunis_DH_genome.v2.0). Variants were then sorted by their predicted effect, and those annotated as of HIGH impact were selected for manual inspection. Genes affected by HIGH impact variants were selected and their predicted functional analysis was retrieved from genome-related files available at GDR, namely GO assignments, IPR assignments and KEGG pathways, using a custom python script.

### Transposable elements

Genome-wide analysis of Transposable elements (TE) insertions for each sample was performed using PoPoolationTE2 [53] and Retroseq [54] softwares through the McClintock pipeline [55]. In detail, a TE consensus library for *P. communis* was downloaded from URGIT RepetDB (http://urgi.versailles.inra.fr/repetdb/begin.do#search?taxonGroup=23211). Then, McClintock was used with the —make_annotations argument to create the annotation and taxonomy files, followed by TE insertions (TEI) discovery for each sample individually using the PoPoolationte2 and Retroseq methods. The individual result files for each sample were merged and then used to count the number of TEs per class and per number of clones where it was discovered, using in-house python scripts. A given TEI was only included for the final results dataset if it was newly discovered (non-reference) and confirmed by both forward and reverse reads in the case of PoPoolationTE2 (non-redundant). Since new insertions are only pinpointed (i.e. the spawn of the TEI is only 1 bp), the overlap of TEI among clones, or the calculation of which insertions were shared by *N* clones, was achieved as follows: if the same TE was present in more than one clone, in the same chromosome, it was considered to be shared between that many clones if the pinpoint positions of those TEs were less than 10 Kbp apart.

## Acknowledgements

The authors would like to thank Ana Elisabete Pires and Catarina Ginja from the Research Centre in Biodiversity and Genetic Resources (CIBIO) for their support on the design and discussion of this work.

## Authors contribution

OS performed the bioinformatics analysis and wrote the manuscript. JG and FS performed sample collection, DNA extraction and polymorphism validation. RS, MLS, FS, PS and JM reviewed the manuscript.

## Data availability

The raw sequences used in this article were deposited at the NCBI SRA database and are available under the BioProject 780683 (http://www.ncbi.nlm.nih.gov/bioproject/780683). The variants dataset was submitted to the Genome Database for Rosaceae (https://www.rosaceae.org/publication_datasets) and are available under the accession number tfGDR1058

## Conflicts of interest

The authors declare no conflict of interest.

## Supplementary data

Supplementary data is available at *Horticulture Research Journal* online.

## Supplementary Material

Serra_et_al_suplementary_uhac111

Table_S5_uhac111

## References

[1] FAOSTAT . http://www.fao.org/faostat/en/#data/QCL 2021.

[2] Asanidze Z, Akhalkatsi M, Gvritishvili M. Comparative morphometric study and relationships between the Caucasian species of wild pear (*Pyrus* spp.) and local cultivars in Georgia. Flora-Morphol Distrib Funct Ecol Plants. 2011;206:974–86.

[3] Silva GJ, Souza TM, Barbieri RL et al. Origin, domestication, and dispersing of pear (*Pyrus* spp.). Adv Agric. 2014;2014:e541097.

[4] Quinet M, Wesel J-P. Botany and Taxonomy of Pear. In: Korban SS, ed. The Pear Genome. Compendium of Plant Genomes. Springer, Cham, 2019,1–33.

[5] Jiang S, Zheng X, Yu P et al.. Primitive genepools of Asian pears and their complex hybrid origins inferred from fluorescent sequence-specific amplification polymorphism (SSAP) markers based on LTR retrotransposons. PLoS One. 2016;11:e0149192. 26871452 10.1371/journal.pone.0149192 PMC4752223

[6] Katayama H, Amo H, Wuyun T et al.. Genetic structure and diversity of the wild Ussurian pear in East Asia. Breed Sci. 2016;66:90–9. 27069394 10.1270/jsbbs.66.90 PMC4780806

[7] Xue L, Liu Q, Qin M et al.. Genetic variation and population structure of “Zangli” pear landraces in Tibet revealed by SSR markers. Tree Genet Genomes. 2017;13:26.

[8] Zong Y, Sun P, Yue X et al.. Variation in microsatellite loci reveals a natural boundary of genetic differentiation among Pyrus betulaefolia populations in northern China. J Am Soc Hortic Sci. 2017;142:319–29.

[9] Volk GM, Richards CM, Henk AD et al.. Diversity of wild Pyrus communis based on microsatellite analyses. J Am Soc Hortic Sci. 2006;131:408–17.

[10] Wolko Ł, Antkowiak W, Lenartowicz E et al.. Genetic diversity of European pear cultivars (*Pyrus communis L.*) and wild pear (*Pyrus pyraster (L.) Burgsd.*) inferred from microsatellite markers analysis. Genet Resour Crop Evol. 2010;57:801–6.

[11] Wolko Ł, Bocianowski J, Antkowiak W et al.. Genetic diversity and population structure of wild pear (*Pyrus pyraster (L.) Burgsd*.) in Poland. Open Life Sci. 2015;10:19–29.

[12] Zheng X, Cai D, Potter D et al.. Phylogeny and evolutionary histories of Pyrus L. revealed by phylogenetic trees and networks based on data from multiple DNA sequences. Mol Phylogenet Evol. 2014;80:54–65. 25083939 10.1016/j.ympev.2014.07.009

[13] Wu J, Wang Z, Shi Z et al.. The genome of the pear (Pyrus bretschneideri Rehd.). Genome Res. 2013;23:396–408. 23149293 10.1101/gr.144311.112 PMC3561880

[14] Chagné D, Crowhurst RN, Pindo M et al.. The draft genome sequence of European pear (Pyrus communis L. ‘Bartlett’). PLoS One. 2014;9:e92644. 24699266 10.1371/journal.pone.0092644 PMC3974708

[15] Linsmith G, Rombauts S, Montanari S et al.. Pseudo-chromosome-length genome assembly of a double haploid ‘Bartlett’ pear (Pyrus communis L.). GigaScience. 2019;8:giz138. 31816089 10.1093/gigascience/giz138 PMC6901071

[16] Xue H, Wang S, Yao J-L et al.. Chromosome level high-density integrated genetic maps improve the Pyrus bretschneideri ‘DangshanSuli’ v1.0 genome. BMC Genomics. 2018;19:833. 30463521 10.1186/s12864-018-5224-6 PMC6249763

[17] Shirasawa K, Itai A, Isobe S. Chromosome-scale genome assembly of Japanese pear (Pyrus pyrifolia) variety ‘Nijisseiki’. DNA Res. 2021;28:dsab001. 33638981 10.1093/dnares/dsab001 PMC8092371

[18] Ou C, Wang F, Wang J et al.. A de novo genome assembly of the dwarfing pear rootstock Zhongai 1. Sci Data. 2019;6:281. 31767847 10.1038/s41597-019-0291-3 PMC6877535

[19] Dong X, Wang Z, Tian L et al.. De novo assembly of a wild pear (Pyrus betuleafolia) genome. Plant Biotechnol J. 2020;18:581–95. 31368610 10.1111/pbi.13226 PMC6953202

[20] Wu J, Wang Y, Xu J et al.. Diversification and independent domestication of Asian and European pears. Genome Biol. 2018;19:77. 29890997 10.1186/s13059-018-1452-y PMC5996476

[21] Gabay G, Dahan Y, Izhaki Y et al.. High-resolution genetic linkage map of European pear (Pyrus communis) and QTL fine-mapping of vegetative budbreak time. BMC Plant Biol. 2018;18:175. 30165824 10.1186/s12870-018-1386-2 PMC6117884

[22] Oh S, Oh Y, Kim K et al.. Construction of high-resolution genetic linkage map in pear pseudo-BC1 ((Pyrus pyrifolia × P. communis) × P. pyrifolia) using GBS-SNPs and SSRs. Hortic Environ Biotechnol. 2020;61:745–53.

[23] Kumar S, Kirk C, Deng C et al.. Genotyping-by-sequencing of pear (Pyrus spp.) accessions unravels novel patterns of genetic diversity and selection footprints. Hortic Res. 2017;4:1–10. 10.1038/hortres.2017.15 PMC5389204 28451438

[24] Nishio S, Hayashi T, Shirasawa K et al.. Genome-wide association study of individual sugar content in fruit of Japanese pear (Pyrus spp.). BMC Plant Biol. 2021;21:378. 34399685 10.1186/s12870-021-03130-2 PMC8369641

[25] Zhang M-Y, Xue C, Hu H et al.. Genome-wide association studies provide insights into the genetic determination of fruit traits of pear. Nat Commun. 2021;12:1144. 33602909 10.1038/s41467-021-21378-y PMC7892570

[26] Kumar S, Kirk C, Deng CH et al.. Marker-trait associations and genomic predictions of interspecific pear (Pyrus) fruit characteristics. Sci Rep. 2019;9:9072. 31227781 10.1038/s41598-019-45618-w PMC6588632

[27] Lee HS, Kim GH, Kwon SI et al.. Analysis of ‘Fuji’ apple somatic variants from next-generation sequencing. Genet Mol Res. 2016;15: gmr.15038185. 10.4238/gmr.15038185 27525934

[28] Zhao J, Shen F, Gao Y et al.. Parallel bud mutation sequencing reveals that fruit sugar and acid metabolism potentially influence stress in malus. Int J Mol Sci. 2019;20:5988. 31795097 10.3390/ijms20235988 PMC6928686

[29] Calderón L, Mauri N, Muñoz C et al.. Whole genome resequencing and custom genotyping unveil clonal lineages in ‘Malbec’ grapevines (Vitis vinifera L.). Sci Rep. 2021;11:7775. 33833358 10.1038/s41598-021-87445-y PMC8032709

[30] Roach MJ, Johnson DL, Bohlmann J et al.. Population sequencing reveals clonal diversity and ancestral inbreeding in the grapevine cultivar chardonnay. PLoS Genet. 2018;14:e1007807. 30458008 10.1371/journal.pgen.1007807 PMC6279053

[31] Gambino G, Molin AD, Boccacci P et al.. Whole-genome sequencing and SNV genotyping of ‘Nebbiolo’ (Vitis vinifera L.) clones. Sci Rep. 2017;7:17294. 29229917 10.1038/s41598-017-17405-y PMC5725591

[32] Vondras AM, Minio A, Blanco-Ulate B et al.. The genomic diversification of grapevine clones. BMC Genomics. 2019;20:972. 31830913 10.1186/s12864-019-6211-2 PMC6907202

[33] Queiroz Á, Guimarães JB, Sánchez C et al.. Genetic diversity and structure of the Portuguese pear (Pyrus communis L.) germplasm. Sustainability. 2019;11:5340.

[34] Silva JM, Barba NG, Barros MT et al. ‘Rocha’, the pear from Portugal. Acta Hortic. 2005;671:219–22.

[35] Martin M . Cutadapt removes adapter sequences from high-throughput sequencing reads. EMBnet j. 2011;17:10–2.

[36] Andrew, S. FastQC A Quality Control tool for High Throughput Sequence Data. (2010).

[37] Li H . Aligning sequence reads, clone sequences and assembly contigs with BWA-MEM. arXiv:1303.3997v2 [q-bio.GN].

[38] Li H, Handsaker B, Wysoker A et al.. The sequence alignment/map format and SAMtools. Bioinformatics. 2009;25:2078–9. 19505943 10.1093/bioinformatics/btp352 PMC2723002

[39] Picard toolkit. Broad Institute, GitHub repository (2019).

[40] Okonechnikov K, Conesa A, García-Alcalde F. Qualimap 2: advanced multi-sample quality control for high-throughput sequencing data. Bioinformatics. 2016;32:292–4. 26428292 10.1093/bioinformatics/btv566 PMC4708105

[41] Poplin R, Ruano-Rubio V, DePristo MA et al.. Scaling accurate genetic variant discovery to tens of thousands of samples. bioRxiv 201178. 2018.

[42] Li H . A statistical framework for SNP calling, mutation discovery, association mapping and population genetical parameter estimation from sequencing data. Bioinformatics. 2011;27:2987–93. 21903627 10.1093/bioinformatics/btr509 PMC3198575

[43] Danecek P, Auton A, Abecasis G et al.. The variant call format and VCFtools. Bioinformatics. 2011;27:2156–8. 21653522 10.1093/bioinformatics/btr330 PMC3137218

[44] Untergasser A, Nijveen H, Rao X et al.. Primer3Plus, an enhanced web interface to Primer3. Nucleic Acids Res. 2007;35:W71–4. 17485472 10.1093/nar/gkm306 PMC1933133

[45] R Core Team R:A Language and Environment for Statistical Computing. R Foundation for Statistical Computing, 2021.

[46] Pfeifer B, Wittelsbürger U, Ramos-Onsins SE et al. PopGenome: an efficient Swiss army knife for population genomic analyses in R. Mol Biol Evol. 2014;31:1929–36. 24739305 10.1093/molbev/msu136 PMC4069620

[47] Manichaikul A, Mychaleckyj JC, Rich SS et al.. Robust relationship inference in genome-wide association studies. Bioinformatics. 2010;26:2867–73. 20926424 10.1093/bioinformatics/btq559 PMC3025716

[48] Gogarten SM, Sofer T, Chen H et al.. Genetic association testing using the GENESIS R/Bioconductor package. Bioinformatics. 2019;35:5346–8. 31329242 10.1093/bioinformatics/btz567 PMC7904076

[49] Purcell S, Neale B, Todd-Brown K et al. PLINK: a tool set for whole-genome association and population-based linkage analyses. Am J Hum Genet. 2007;81:559–75. 17701901 10.1086/519795 PMC1950838

[50] Wickham H . ggplot2: Elegant Graphics for Data Analysis. Springer-Verlag, New York, 2009.

[51] Bailey TL, Boden M, Buske FA et al. MEME SUITE: tools for motif discovery and searching. Nucleic Acids Res. 2009;37:W202–8. 19458158 10.1093/nar/gkp335 PMC2703892

[52] Cingolani P, Platts A, Wang LL et al.. A program for annotating and predicting the effects of single nucleotide polymorphisms, SnpEff: SNPs in the genome of Drosophila melanogaster strain w1118; iso-2; iso-3. Fly (Austin). 2012;6:80–92. 22728672 10.4161/fly.19695 PMC3679285

[53] Kofler R, Gómez-Sánchez D, Schlötterer C. PoPoolationTE2: comparative population genomics of transposable elements using Pool-Seq. Mol Biol Evol. 2016;33:2759–64. 27486221 10.1093/molbev/msw137 PMC5026257

[54] Keane TM, Wong K, Adams DJ. RetroSeq: transposable element discovery from next-generation sequencing data. Bioinformatics. 2013;29:389–90. 23233656 10.1093/bioinformatics/bts697 PMC3562067

[55] Nelson MG, Linheiro RS, Bergman CM. McClintock: an integrated pipeline for detecting transposable element insertions in whole-genome shotgun sequencing data. G3 (Bethesda). 2017;7:2763–78. 28637810 10.1534/g3.117.043893 PMC5555480

[56] Montanari S, Bianco L, Allen BJ et al.. Development of a highly efficient AxiomTM 70 K SNP array for Pyrus and evaluation for high-density mapping and germplasm characterization. BMC Genomics. 2019;20:331. 31046664 10.1186/s12864-019-5712-3 PMC6498479

[57] Montanari S, Postman J, Bassil NV et al.. Reconstruction of the largest pedigree network for pear cultivars and evaluation of the genetic diversity of the USDA-ARS National *Pyrus* Collection. G3 (Bethesda). 2020;10:3285–97. 32675069 10.1534/g3.120.401327 PMC7466967

[58] Dong X, Hong Z, Sivaramakrishnan M et al.. Callose synthase (CalS5) is required for exine formation during microgametogenesis and for pollen viability in Arabidopsis. Plant J. 2005;42:315–28. 15842618 10.1111/j.1365-313X.2005.02379.x

[59] Nishikawa S, Zinkl GM, Swanson RJ et al.. Callose (beta-1,3 glucan) is essential for Arabidopsis pollen wall patterning, but not tube growth. BMC Plant Biol. 2005;5:22. 16212660 10.1186/1471-2229-5-22 PMC1274334

[60] Rajaraman J, Douchkov D, Hensel G et al.. An LRR/Malectin receptor-like kinase mediates resistance to non-adapted and adapted powdery mildew fungi in barley and wheat. Front Plant Sci. 2016;7:1836. 28018377 10.3389/fpls.2016.01836 PMC5156707

[61] Wang D, Liang X, Bao Y et al.. A malectin-like receptor kinase regulates cell death and pattern-triggered immunity in soybean. EMBO Rep. 2020;21:e50442. 32924279 10.15252/embr.202050442 PMC7645207

[62] Huck N, Moore JM, Federer M et al.. The Arabidopsis mutant feronia disrupts the female gametophytic control of pollen tube reception. Development. 2003;130:2149–59. 12668629 10.1242/dev.00458

[63] Rotman N, Gourgues M, Guitton A-E et al. A dialogue between the Sirène pathway in Synergids and the fertilization independent seed pathway in the central cell controls male gamete release during double fertilization in Arabidopsis. Mol Plant. 2008;1:659–66. 19825570 10.1093/mp/ssn023

[64] Kou X, Qi K, Qiao X et al. Evolution, expression analysis, and functional verification of Catharanthus roseus RLK1-like kinase (CrRLK1L) family proteins in pear (Pyrus bretchneideri). Genomics. 2017;109:290–301. 28502783 10.1016/j.ygeno.2017.05.003

[65] Jia M, Du P, Ding N et al. Two FERONIA-like receptor kinases regulate apple fruit ripening by modulating ethylene production. Front Plant Sci. 2017;8:1406. 28848599 10.3389/fpls.2017.01406 PMC5554343

[66] Bieluszewski T, Galganski L, Sura W et al. AtEAF1 is a potential platform protein for Arabidopsis NuA4 acetyltransferase complex. BMC Plant Biol. 2015;15:75. 25849764 10.1186/s12870-015-0461-1 PMC4358907

[67] Malenica N, Maletic E, Simon S et al. Grapevine variety determination from herbarium and archeological specimens. Acta Hortic. 2014;1046:603–8.

[68] Franco-Zorrilla JM, López-Vidriero I, Carrasco JL et al. DNA-binding specificities of plant transcription factors and their potential to define target genes. Proc Natl Acad Sci U S A. 2014;111:2367–72. 24477691 10.1073/pnas.1316278111 PMC3926073

