# Supplementary material for: Genome-wide clonal variability in European pear “Rocha” using high-throughput sequencing
Source: Hortic Res. 2022 May 17;9:uhac111. doi: 10.1093/hr/uhac111 (PMC10939347; doi:10.1093/hr/uhac111)

**Table S1** - The amount of raw and clean reads, mapped reads and percentage of genome coverage at different thresholds for each ‘Rocha’ accession. MQ - Mapping quality

| **Sample** | **Raw reads** | **Mean depth of raw reads** | **Clean reads** | **Mapped reads (MQ>30)** | **Mean depth of mapped reads** | **Genome coverage (%) >= 1** | **Genome coverage (%) >= 5** | **Genome coverage (%) >= 10** |
| --- | --- | --- | --- | --- | --- | --- | --- | --- |
| PRT 50 | 70998406 | 21.3× | 70426720 | 37091561 | 10.9× | 77.60% | 70.64% | 58.42% |
| PRT 51 | 110091512 | 33.1× | 107129848 | 67335316 | 19.8× | 87.73% | 83.20% | 78.12% |
| PRT 52 | 90061894 | 27.1× | 87819282 | 56263106 | 16.5× | 87.43% | 82.38% | 75.18% |
| PRT 53 | 84849262 | 25.5× | 82469480 | 53039900 | 15.6× | 87.20% | 81.84% | 73.27% |
| PRT 55 | 90690234 | 27.3× | 88332760 | 55844823 | 16.4× | 87.21% | 82.05% | 74.14% |
| PRT 56 | 75755236 | 22.8× | 73906448 | 47876742 | 14.1× | 86.99% | 81.16% | 70.11% |
| PRT 57 | 96006234 | 28.9× | 93863322 | 59154756 | 17.4× | 87.36% | 82.50% | 75.86% |
| PRT 58 | 92426866 | 27.8× | 90294602 | 58977911 | 17.3× | 87.35% | 82.52% | 76.11% |

**Table S2** - The mean depth, the number of variants excluded due to quality filtering, the classification of variants as homozygous or heterozygous and the frequency of variants per 1 Kbp, for each ‘Rocha’ pear accession. **A** - SNP data; **B** - indel data.

**A**

| **Sample** | **SNPs Filtered out** | **Heterozygous SNPs** | **Homozygous SNPs** | **Total SNPs** | **SNP frequency per 10^^3^ bp** |
| --- | --- | --- | --- | --- | --- |
| PRT 50 | 566515 | 816525 | 1605746 | 2422271 | 6.88 |
| PRT 51 | 40278 | 1089041 | 1936449 | 3025490 | 7.3 |
| PRT 52 | 67283 | 1076539 | 1920990 | 2997529 | 7.3 |
| PRT 53 | 87240 | 1069053 | 1906927 | 2975980 | 7.3 |
| PRT 55 | 80773 | 1071175 | 1910953 | 2982128 | 7.29 |
| PRT 56 | 118477 | 1055599 | 1886136 | 2941735 | 7.27 |
| PRT 57 | 58592 | 1082252 | 1924325 | 3006577 | 7.31 |
| PRT 58 | 61168 | 1079851 | 1923347 | 3003198 | 7.3 |

**B**

| **Sample** | **Indels Filtered out** | **Heterozygous Indels** | **Homozygous Indels** | **Total Indels** | **Indel frequency per 10^^3^ bp** |
| --- | --- | --- | --- | --- | --- |
| PRT 50 | 72724 | 96848 | 144145 | 240993 | 0.68 |
| PRT 51 | 5464 | 139165 | 188217 | 327382 | 0.79 |
| PRT 52 | 9249 | 136796 | 186403 | 323199 | 0.79 |
| PRT 53 | 11723 | 135509 | 184821 | 320330 | 0.79 |
| PRT 55 | 11090 | 135441 | 185467 | 320908 | 0.78 |
| PRT 56 | 15313 | 133328 | 182759 | 316087 | 0.78 |
| PRT 57 | 7313 | 138251 | 186999 | 325250 | 0.79 |
| PRT 58 | 7918 | 137444 | 186669 | 324113 | 0.79 |

**Table S3** - The amount of SNP positions (A) and Indel positions (B), per chromosome, that passed filter criteria for a given number of ‘Rocha’ pear samples.

**A**

| **Chromosome** | **Positions available in all samples** | **Positions available in 7 samples** | **Positions available in 6 samples** | **Positions available in 5 samples** | **Positions available in 4 samples** | **Total** |
| --- | --- | --- | --- | --- | --- | --- |
| Chr1 | 94041 | 14820 | 3434 | 1886 | 1242 | 115423 |
| Chr2 | 128094 | 25518 | 6211 | 3292 | 2339 | 165454 |
| Chr3 | 72981 | 10365 | 2388 | 1325 | 1086 | 88145 |
| Chr4 | 134983 | 27493 | 6608 | 3799 | 2882 | 175765 |
| Chr5 | 186560 | 40767 | 10507 | 5994 | 4482 | 248310 |
| Chr6 | 162590 | 34633 | 8757 | 4580 | 3296 | 213856 |
| Chr7 | 161873 | 36400 | 9644 | 5081 | 3848 | 216846 |
| Chr8 | 119120 | 25385 | 6188 | 3203 | 2510 | 156406 |
| Chr9 | 151410 | 28875 | 5976 | 3591 | 2743 | 192595 |
| Chr10 | 229740 | 48563 | 11716 | 6268 | 4757 | 301044 |
| Chr11 | 128665 | 20911 | 4521 | 2455 | 1898 | 158450 |
| Chr12 | 65919 | 8613 | 1574 | 817 | 598 | 77521 |
| Chr13 | 136665 | 32175 | 7787 | 4070 | 3153 | 183850 |
| Chr14 | 137752 | 28706 | 6554 | 3614 | 2637 | 179263 |
| Chr15 | 225099 | 41816 | 9864 | 5308 | 3763 | 285850 |
| Chr16 | 135161 | 30017 | 6942 | 3808 | 3058 | 178986 |
| Chr17 | 114789 | 23999 | 6070 | 3001 | 2086 | 149945 |
| Total | 2385442 | 479056 | 114741 | 62092 | 46378 | 3087709 |

**Table S3** - (*Continued*)

**B**

| **Chromosome** | **Positions present in all samples** | **Positions present in 7 samples** | **Positions present in 6 samples** | **Positions present in 5 samples** | **Positions present in 4 samples** | **Total** |
| --- | --- | --- | --- | --- | --- | --- |
| Chr1 | 10989 | 2446 | 587 | 289 | 215 | 14526 |
| Chr2 | 13699 | 3422 | 819 | 437 | 309 | 18686 |
| Chr3 | 10081 | 1938 | 435 | 223 | 134 | 12811 |
| Chr4 | 13608 | 3262 | 771 | 438 | 353 | 18432 |
| Chr5 | 18839 | 5008 | 1373 | 762 | 594 | 26576 |
| Chr6 | 15423 | 3947 | 1089 | 611 | 383 | 21453 |
| Chr7 | 16757 | 4394 | 1232 | 656 | 509 | 23548 |
| Chr8 | 13159 | 3366 | 929 | 456 | 362 | 18272 |
| Chr9 | 14509 | 3666 | 850 | 484 | 383 | 19892 |
| Chr10 | 22313 | 6056 | 1409 | 807 | 623 | 31208 |
| Chr11 | 14667 | 3356 | 671 | 365 | 266 | 19325 |
| Chr12 | 9833 | 1907 | 390 | 197 | 136 | 12463 |
| Chr13 | 12484 | 3350 | 798 | 428 | 362 | 17422 |
| Chr14 | 13793 | 3464 | 839 | 475 | 378 | 18949 |
| Chr15 | 22856 | 5474 | 1301 | 707 | 544 | 30882 |
| Chr16 | 12587 | 3240 | 726 | 407 | 307 | 17267 |
| Chr17 | 13070 | 3321 | 806 | 407 | 312 | 17916 |
| Total | 248667 | 61617 | 15025 | 8149 | 6170 | 339628 |

**Table S4 -** The motifs discovered in “High mutation rate” (>5 SNPs/Kbp) and “Very high mutation rate” (> 10 SNPs/Kbp) regions, and their match with known databases.

| **Class of region** | **Motif** | **Match** | **Database match** |
| --- | --- | --- | --- |
| High mutation rate | AAAAATCTA | RVE1  CCA1  RVE1_2 | franco-zorrilla2014 |
| High mutation rate | AAATTTAAA | CPP_tnt.TSO1_col_a_m1  CPP_tnt.SOL1_col_m1  CPP_tnt.AT2G20110_col_a_m1  CPP_tnt.TCX2_colamp_a_m1  CPP_tnt.SOL1_colamp_a_m1  CPP_tnt.TCX2_col_a_m1  CPP_tnt.AT2G20110_colamp_a_m1 | Omalley2016 |
| High mutation rate | AATCACGTG | bHLH_tnt.bHLH74_colamp_a_m1  bHLH_tnt.BIM1_col_a_m1 | Omalley2016 |
| Very high mutation rate | ACTACTTCACCAADT | NAC_tnt.NTM1_col_a_m1  C2H2_tnt.At2g48100_col_b_m1  MADS_tnt.AGL55_col_a_m1  MADS_tnt.FEM111_col_a_m1  NAC_tnt.NTM2_col_b_m1  NAC_tnt.ANAC103_col_a_m1 | Omalley2016 |
| High mutation rate | ACTCAAAGTTTTCA | CCAATHAP3_tnt.NFYB4_col_a_m1 | Omalley2016 |
| High mutation rate | AGGATCCACTCCC | TCP16 | franco-zorrilla2014 |
| High mutation rate | GACGTCAT | bZIP_tnt.TGA9_colamp_a_m1  bZIP_tnt.TGA4_col_a_m1  bZIP_tnt.TGA9_col_a_m1  bZIP_tnt.TGA3_colamp_a_m1  bZIP_tnt.TGA1_colamp_a_m1  bZIP_tnt.TGA10_col_a_m1  bZIP_tnt.TGA5_col_v3a_m1  bZIP_tnt.TGA10_colamp_a_m1  bZIP_tnt.TGA6_col_a_m1  bZIP_tnt.TGA6_colamp_a_m1  bZIP_tnt.TGA1_col_m1  bZIP_tnt.TGA4_colamp_a_m1  bZIP_tnt.TGA3_col_m1  bZIP_tnt.TGA2_col_m1  bZIP_tnt.bZIP50_col_v31_m1  bZIP_tnt.bZIP50_colamp_v31_m1  bZIP_tnt.TGA2_colamp_v31_m1 | Omalley2016 |
|  |  | TGA2  bZIP60  RAP2.6_3ary  TGA2_2 | franco-zorrilla2014 |
| High mutation rate | GCTCTAGAACMC | HSFC1  HSFB2A_2 | franco-zorrilla2014 |
| High mutation rate | GGCCCACRM | TCP_tnt.At5g08330_col_a_m1  TCP_tnt.At1g72010_colamp_a_m1 | Omalley2016 |
|  |  | TCP15  TCP23 | franco-zorrilla2014 |
| High mutation rate | GGTCAACGACAC | WRKY38  WRKY12  WRKY45 | franco-zorrilla2014 |

**Table S4 -** (*Continued*)

| **Class of region** | **Motif** | **Match** | **Database match** |
| --- | --- | --- | --- |
| High mutation rate | TAAAAAAAAAT | C3H_tnt.EMB1789_col_a_m1  C2C2dof_tnt.OBP3_colamp_a_m1 | Omalley2016 |
| High mutation rate | TAARAAGTCDA | WRKY_tnt.WRKY50_col_a_m1  WRKY_tnt.WRKY45_col_a_m1  WRKY_tnt.WRKY17_colamp_a_m1  WRKY_tnt.WRKY17_col_a_m1  WRKY_tnt.WRKY18_colamp_a_m1  WRKY_tnt.WRKY43_col_a_m1  WRKY_tnt.WRKY7_col_m1  WRKY_tnt.WRKY24_col_a_m1  WRKY_tnt.WRKY40_col_m1  WRKY_tnt.WRKY43_colamp_a_m1  WRKY_tnt.WRKY8_col_m1  WRKY_tnt.WRKY8_colamp_a_m1 | Omalley2016 |
| High mutation rate | TTAAAAAATTAWT | AHL25_3ary | franco-zorrilla2014 |
| High mutation rate | WTTTTAAAAW | CPP_tnt.TSO1_col_a_m1 | Omalley2016 |

**Table S5 -** All HIGH impact variants, caused by polymorphic SNPs or IINDELs within ‘Rocha’ accessions, that were found in this study, their locations, the gene(s) affected and the GO, IPR and KEEG for every gene affected.

*Please see attached file “Table_S5.xlsx”*

**Table S6** - The distribution of non-Reference TE insertions in *N* ‘Rocha’ clones by their Class and Order using both Retroseq (A) and PopoolationTE2 (B) softwares.

**A**

| **Class** | **Order** | **Retroseq** | | | | | | | |
| --- | --- | --- | --- | --- | --- | --- | --- | --- | --- |
| **Class I** | **LINE** | 62 | 18 | 5 | 1 | 5 | 1 | 0 | 0 |
|  | **LTR** | 1693 | 994 | 527 | 312 | 186 | 158 | 131 | 74 |
|  | **SINE** | 46 | 14 | 5 | 5 | 2 | 1 | 1 | 0 |
|  | **SINE** | 5 | 2 | 1 | 1 | 0 | 0 | 2 | 0 |
|  | **LARD** | 7 | 6 | 1 | 1 | 0 | 0 | 0 | 0 |
|  | **?** | 5 | 2 | 0 | 0 | 0 | 0 | 0 | 0 |
|  | **DIRS** | 23 | 3 | 3 | 1 | 2 | 1 | 2 | 0 |
| **Class II** | **Helitron** | 19 | 7 | 6 | 2 | 0 | 1 | 0 | 1 |
|  | **Maverick** | 1 | 0 | 0 | 0 | 0 | 0 | 0 | 0 |
|  | **TIR** | 786 | 370 | 179 | 80 | 68 | 59 | 31 | 12 |
|  | **?** | 285 | 91 | 41 | 18 | 11 | 6 | 3 | 2 |
|  | **MITE** | 516 | 241 | 86 | 65 | 59 | 23 | 24 | 8 |
| **Unclassified** | | 42 | 17 | 8 | 5 | 6 | 2 | 0 | 1 |
| **PotentialHostGene** | | 19 | 0 | 4 | 4 | 0 | 0 | 0 | 0 |
| **Total class I** | | 1841 | 1039 | 542 | 321 | 195 | 161 | 136 | 74 |
| **Total class II** | | 1607 | 709 | 312 | 165 | 138 | 89 | 58 | 23 |
| **Total** | | 3509 | 1765 | 866 | 495 | 339 | 252 | 194 | 98 |
| **Number of ‘Rocha’ clones** | | **1** | **2** | **3** | **4** | **5** | **6** | **7** | **8** |

**B**

| **Class** | **Order** | **PopoolationTE2** | | | | | | | |
| --- | --- | --- | --- | --- | --- | --- | --- | --- | --- |
| **Class I** | **LINE** | 37 | 5 | 4 | 1 | 1 | 1 | 0 | 0 |
|  | **LTR** | 101 | 61 | 32 | 12 | 13 | 5 | 7 | 6 |
|  | **SINE** | 82 | 31 | 16 | 4 | 0 | 2 | 3 | 0 |
|  | **LARD** | 1 | 1 | 0 | 0 | 0 | 1 | 0 | 0 |
|  | **?** | 1 | 1 | 0 | 1 | 0 | 0 | 0 | 0 |
|  | **DIRS** | 2 | 0 | 0 | 0 | 1 | 0 | 0 | 0 |
| **Class II** | **Helitron** | 10 | 8 | 1 | 2 | 1 | 0 | 0 | 0 |
|  | **TIR** | 152 | 65 | 39 | 24 | 7 | 10 | 4 | 0 |
|  | **?** | 159 | 57 | 37 | 20 | 12 | 0 | 1 | 0 |
|  | **MITE** | 36 | 10 | 4 | 6 | 2 | 1 | 1 | 1 |
| **Unclassified** | | 8 | 3 | 1 | 0 | 2 | 2 | 0 | 0 |
| **PotentialHostGene** | | 47 | 14 | 11 | 2 | 1 | 1 | 3 | 0 |
| **Total class I** | | 224 | 99 | 52 | 18 | 15 | 9 | 10 | 6 |
| **Total class II** | | 357 | 140 | 81 | 52 | 22 | 11 | 6 | 1 |
| **Total** | | 636 | 256 | 145 | 72 | 40 | 23 | 19 | 7 |
| **Number of ‘Rocha’ clones** | | **1** | **2** | **3** | **4** | **5** | **6** | **7** | **8** |

**Table S7** – The validation of SNP and INDEL variants, based on the expected genotype of clone PRT 52 from *in silico* vs sanger sequencing in the lab.

| **Chromosome** | **Position** | **Type of variant** | **Reference allele** | **Alternative allele** | ***In silico* genotype** | **Primer F** | **Primer R** | **Sanger sequencing genotype** |
| --- | --- | --- | --- | --- | --- | --- | --- | --- |
| Chr1 | 19794046 | SNP | G | A | G/A | AAACGTGAGTTAGGCCGGTA | GACACCTACAAAACACACATGTCA | *Multiple fragments* |
| Chr2 | 7538077 | SNP | C | T | T/T | GGAAAGGGTAAAGGAGGAGGTA | ATTTGCATTTCCTCAAGGGTTT | *No primer amplification* |
| Chr2 | 17558988 | SNP | C | T | T/T | TGGCTACTTAATTTGCACACAGA | ACAAGGCAAGTGCATGTCATTA | T/T |
| Chr5 | 2169970 | SNP | T | A | A/A | CCATCTACACGATAGGACACGA | ATTCGTCCATTTGACCACATTT | A/A |
| Chr8 | 12297150 | SNP | G | A | G/A | AAAGATGAAGGGCATTTTTGTC | ACTGCAAATTGTCCCATAAGGT | G/A |
| Chr9 | 17707800 | SNP | T | C | T/C | GCTACTGAGCGCGTAAGAAGAT | ACATAGTTGCTGCACTGTTGCT | C/C |
| Chr13 | 5712782 | SNP | T | C | T/C | AGATTGCATCAGTCGTGTCATC | TAAGCACCCACTCTACACCACA | T/C |
| Chr13 | 14499495 | SNP | C | T | T/T | CAAGACTATATGCGCCAATCAA | ATAAGTAAGGCCATTGCTGGAA | T/T |
| Chr17 | 10025310 | SNP | G | A | G/A | TCCTGTTTACTCGAGCATCTCA | GTTGTACTTGGCCATGACAGTG | G/G |
| Chr1 | 16673685 | INDEL | GTAGA | G | GTAGA/G | CTACCCCTTTCTTTGTGATTGC | CATCTTCTCAGCCTCCTCAAAT | *No primer amplification* |
| Chr2 | 11807726 | INDEL | C | CTTTG | C/CTTTG | CCAACTTGATGCTATCCTCTCC | TGGTTGAGATGGTGAAAGATTG | C/CTTTG |
| Chr7 | 24229992 | INDEL | TG | T | T/T | TCTCTCTCTTCTTTCATCTCATTCTG | TTAGTTGAAGACGGAGGCAAAT | *No primer amplification* |
| Chr11 | 24644046 | INDEL | TCC | T | T/T | ACCATGCTCTCGGTATGAGATT | AACCAAGATTGTCCGCTAAGAC | T/T |

**Figure S1 –** Geographical representation of the original ‘Rocha’ Tree near Sintra, and the location of the orchards were each of the eight clones used in this study was first identified. On the left, the map of Portugal. On the right, close-up of the west (“Oeste”) region.

**
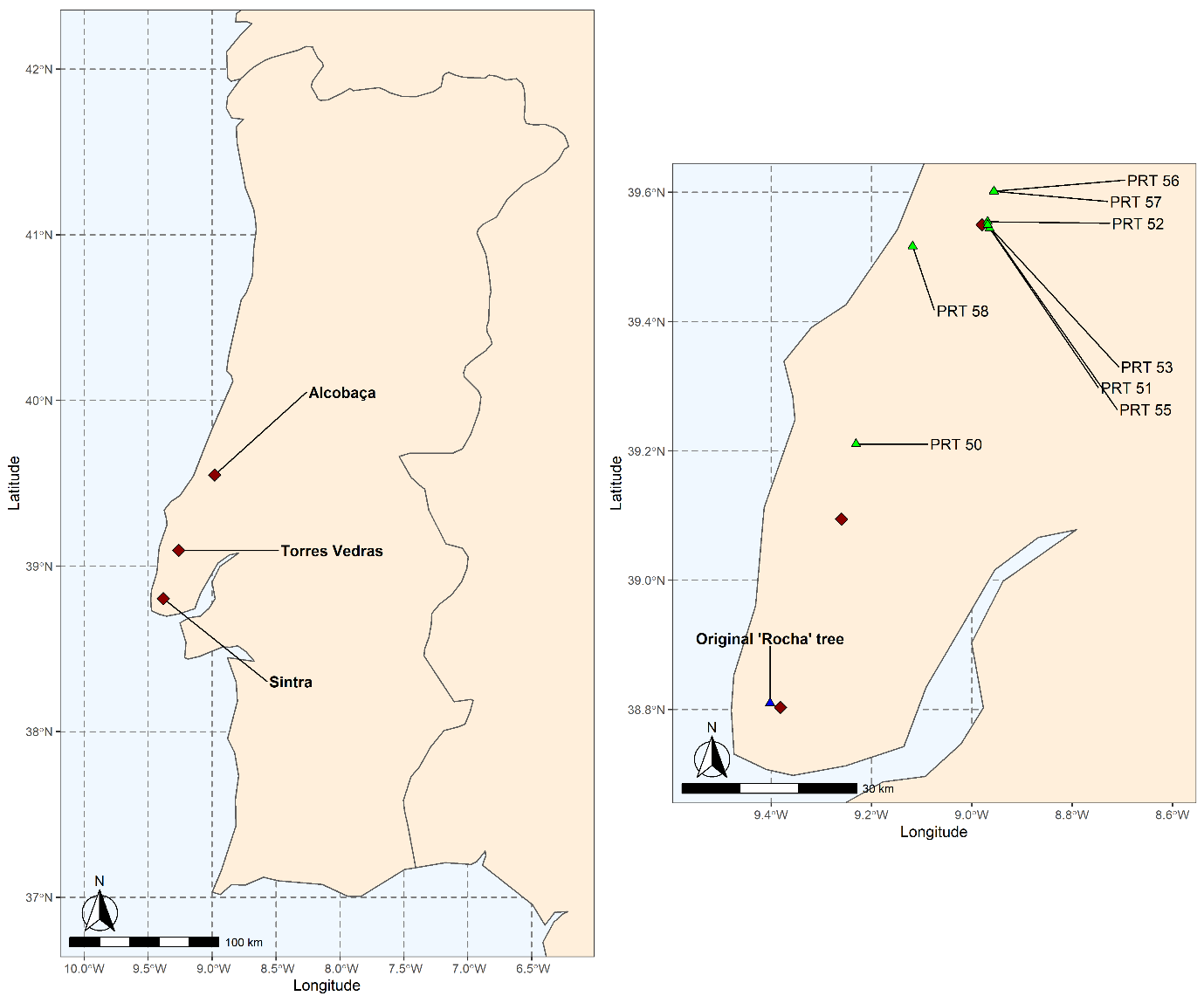
**


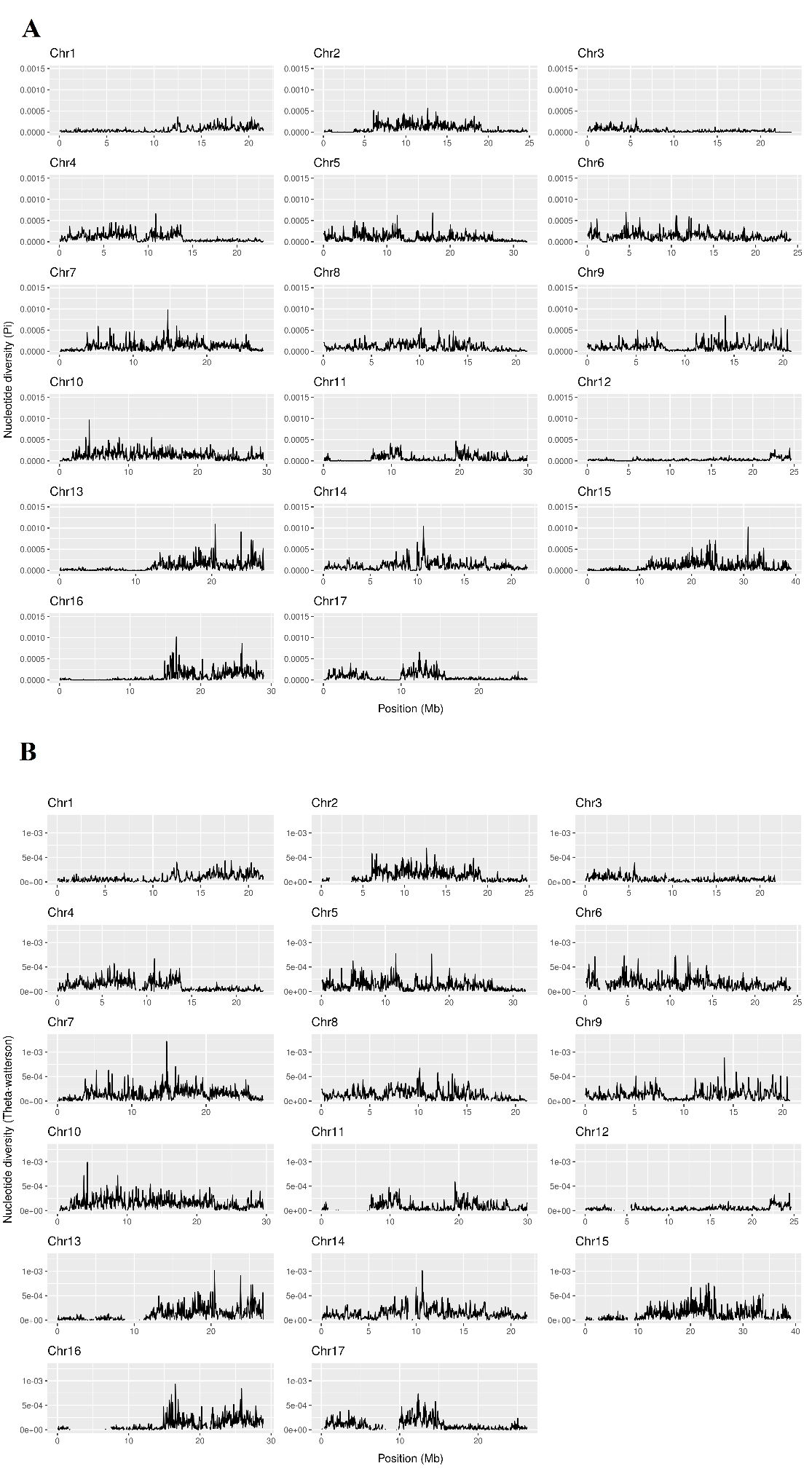
**Figure S2 -** Nucleotide diversity within ‘Rocha’ samples, per chromosome, using 50 Kbp windows. A - Nucleotide diversity (π); B - Nucleotide diversity (θ_w_).

**
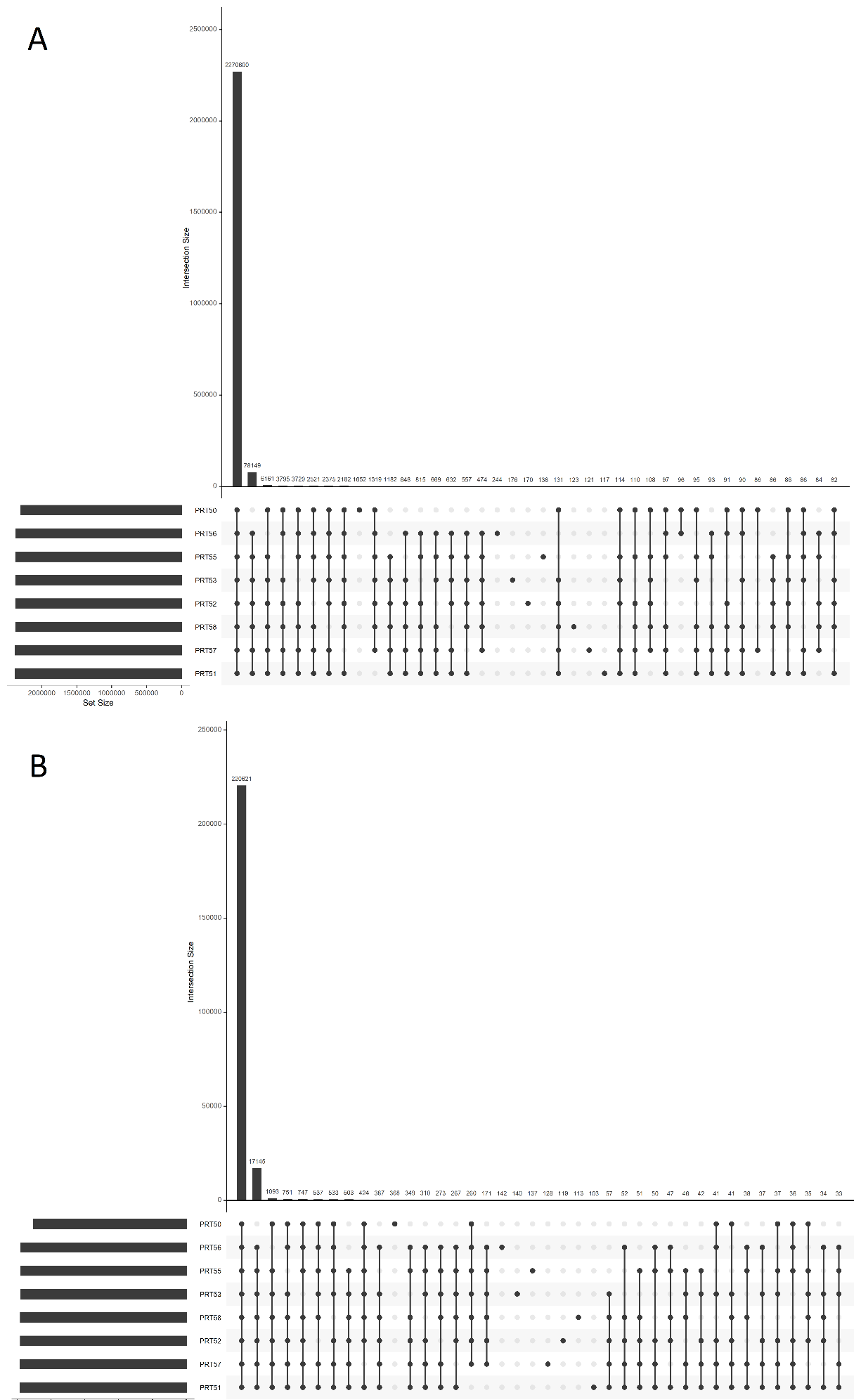
Figure S3 –** The distribution of all SNPs (A) and INDELs (B) from ‘Rocha’ vs ‘Bartlett’ (vertical bars) and how they are shared between clones (bottom of each graph). Connected dots represent variants shared by those clones.

**Figure S4** – Kinship coefficient among ‘Rocha’ accessions. Negative kinship coefficient estimations that indicate unrelated relationships between individuals (the case of outroup PRT11 - ‘Carapinheira’) were converted to zero for visualization purposes.


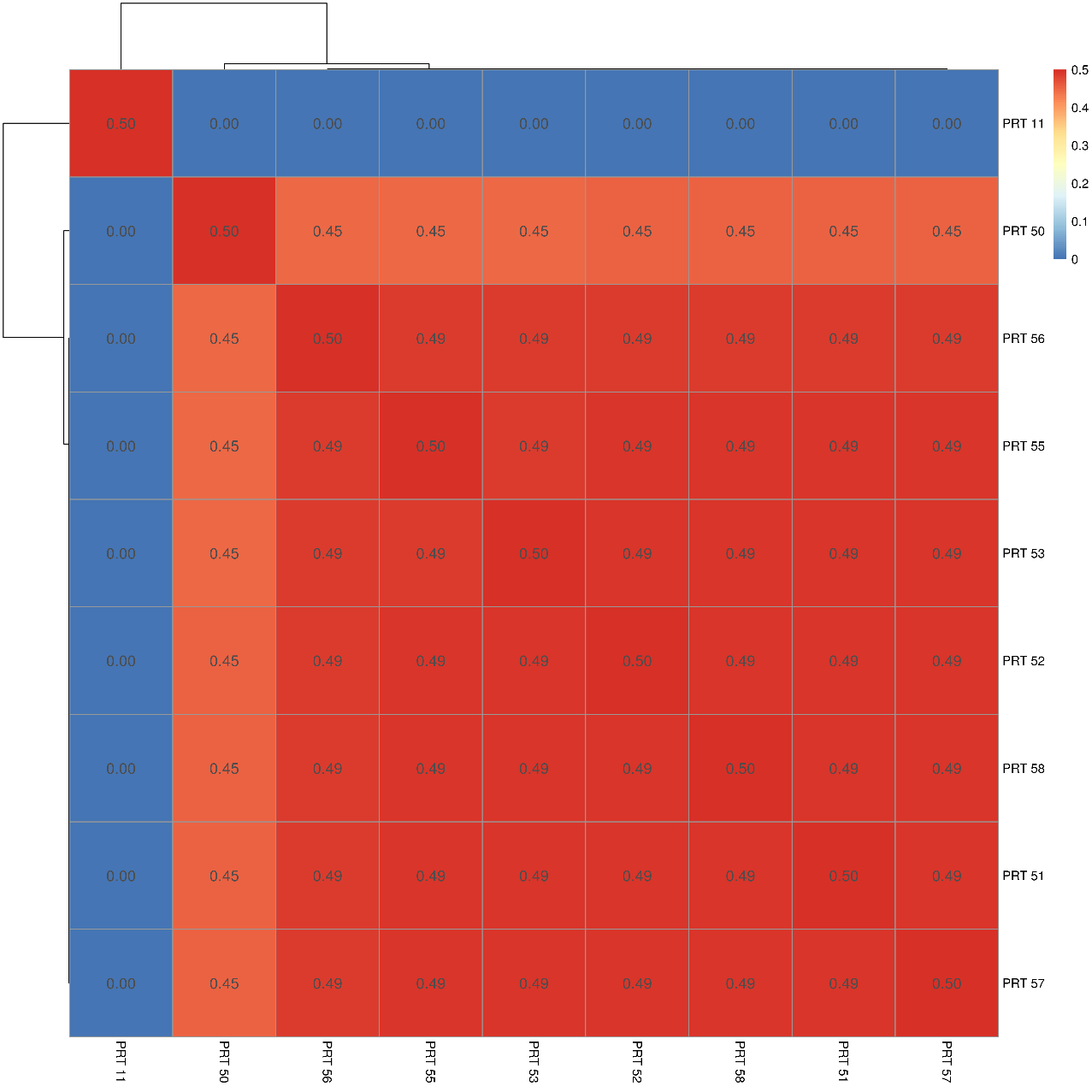


**Figure S5 –** Top five DNA motifs discovered in 1 Kbp regions with A) high levels of mutations (more than 5 mutations) and B) very high levels of mutations (more than 10 mutations).

**
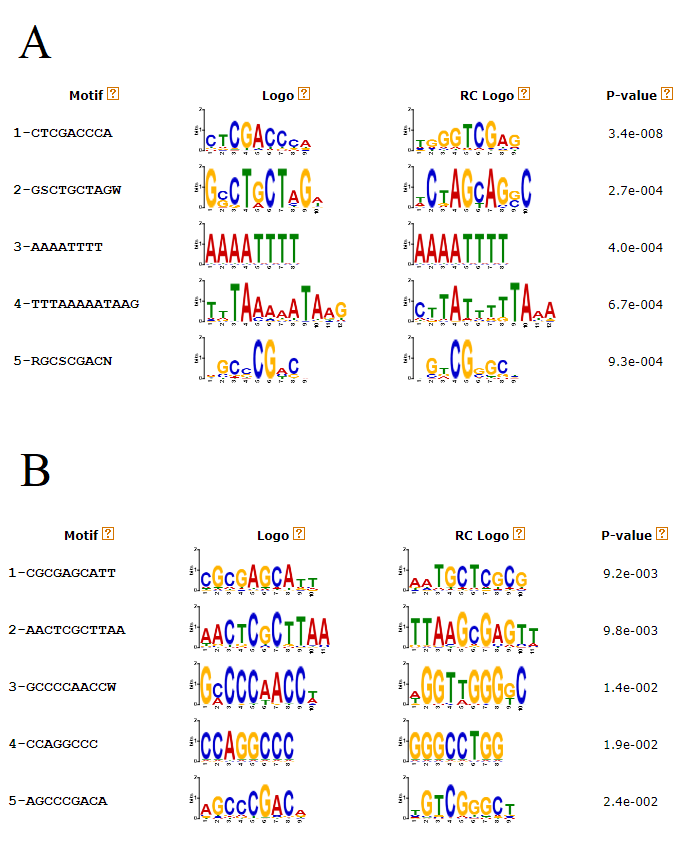
**

**Figure S6** - The amount of non-reference TE insertions per ‘Rocha’ sample at avery Mbp, using A) Retroseq software and B) PoPoolationTE2 software.


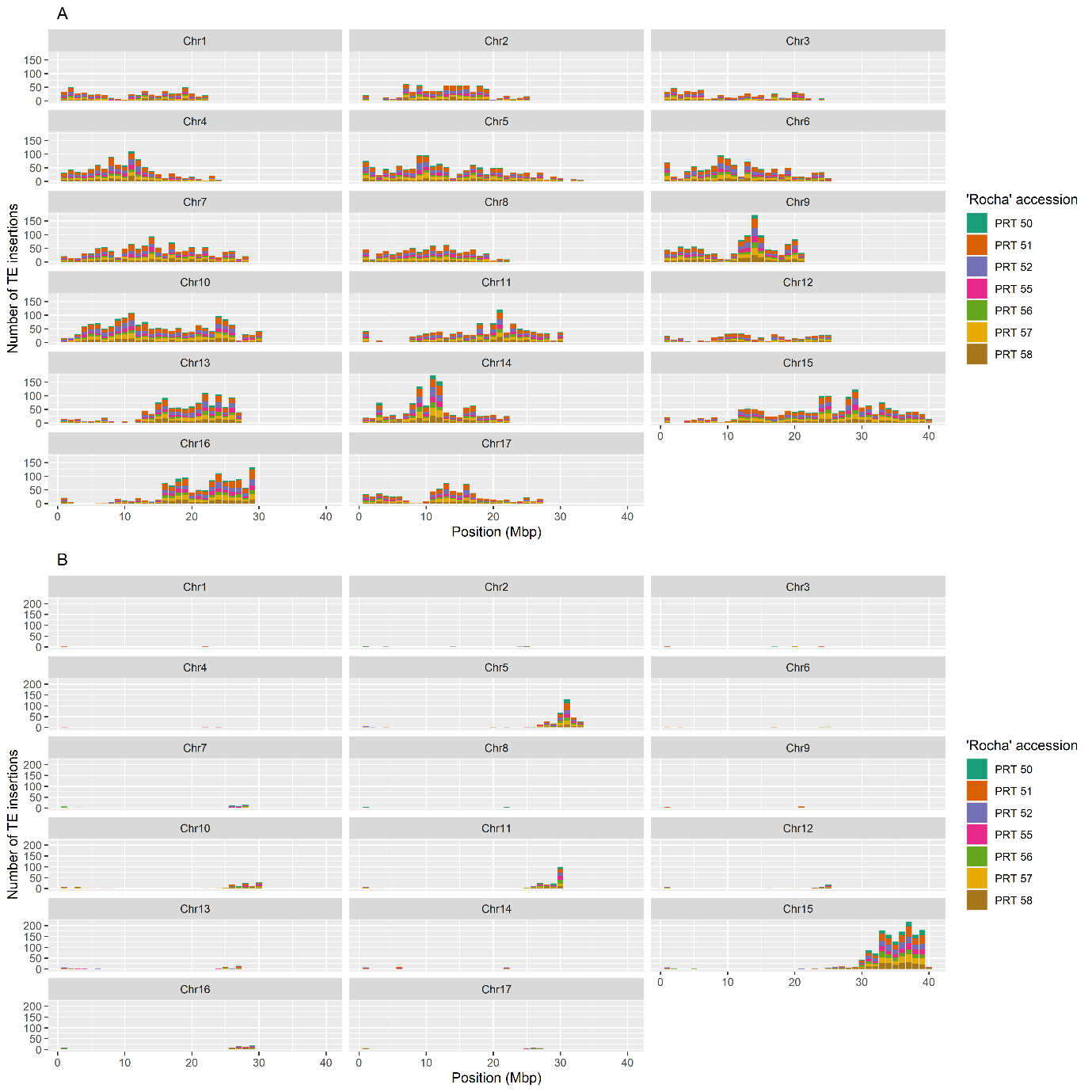

Supplement: Serra_et_al_suplementary_uhac111 [file serra_et_al_suplementary_uhac111.docx]
